# Equity by design principles for digital health interventions

**DOI:** 10.1186/s12939-025-02645-6

**Published:** 2025-10-14

**Authors:** Laura Bitomsky, Marcia Nißen, Tobias Kowatsch

**Affiliations:** 1 Centre for Digital Health Interventions, School of Medicine, University of St. Gallen, St. Gallen, Switzerland; 2 Centre for Digital Health Interventions, Institute for Implementation Science in Health Care, University of Zurich, Zurich, Switzerland; 3 Centre for Digital Health Interventions, Department of Management, Technology, and Economics, ETH Zürich, Zurich, Switzerland

**Keywords:** Health equity, Digital health interventions, Design principles

## Abstract

**Background:**

Despite significant progress in the past decade, health disparities persist. Digital health interventions (DHIs) offer a transformative opportunity to advance health equity but may also exacerbate the digital divide if equity considerations are not embedded from the onset. While there is broad consensus on the importance of equity-centered design, a critical gap re-mains in the form of actionable guidance for both research and practice. Thus, this study aims to develop equity by design principles for DHIs.

**Methods:**

We first synthesized existing scientific knowledge by assessing 42 articles/guidelines and formulated an initial set of 26 actionable, evidence-based design principles for DHIs (July through October 2024). We then conducted three semi-structured expert interviews to refine these principles (November 2024 through January 2025). We finally facilitated end-user workshops with two DHI providers to assess and finalize the design principles with respect to practical relevance and applicability (January through March 2025).

**Results:**

We identified 25 equity by design principles, 15 targeting DHIs, and 10 the organizational context in which DHIs are developed. The DHI-specific principles were categorized according to key process stages: *needs assessment*, *design and development*, *implementation*, and *evaluation and dissemination*. The organizational context principles were grouped into four domains: *strategy*, *people*, *processes and structures*, and *partnerships and advocacy*. We further challenged the principles real-world applicability, identifying three overarching challenges that hinder their successful implementation.

**Conclusions:**

The study underscores the necessity of moving beyond DHI-specific design considerations to address health inequities in digital health. By adopting these design principles, digital health companies can embed equity as a core strategic priority, actively contribute to reducing health disparities, and foster a more inclusive healthcare ecosystem.

**Supplementary Information:**

The online version contains supplementary material available at 10.1186/s12939-025-02645-6.

## Introduction

Despite considerable progress in the past decade, health disparities continue to persist globally. Marginalized populations – such as individuals with low socioeconomic status, racial and ethnic minorities, sexual and gender minorities, and those living with disabilities – continue to face disproportionately elevated risks of non-communicable diseases compared to non-marginalized populations [1–7]. For instance, women spend 25% more time in their lives in poor health compared to men [8]. These populations not only face a heightened burden of disease but additionally face systematic barriers to healthcare access or receive lower quality care [9–12]. Such systematic inequities further exacerbate poor health outcomes and reinforce broader social injustices [13–15]. This presents a societal and public health concern and carries significant economic implications. For instance, racial health disparities contribute to an estimated $93 billion in excess annual healthcare costs in the United States [16], productivity losses due to disability-adjusted life years across Africa amounted to Int$ 2.4 trillion in 2015 [17], and addressing global inequities in women’s health could yield an economic benefit of $1 trillion [8]. Tackling these disparities is critical to achieving equitable healthcare and enhancing overall population health.

Digital health interventions (DHIs) promise a transformative opportunity to advance health equity by addressing longstanding obstacles to care [18, 19]. DHIs leverage various information and communication technologies to collect, store, share, and analyze health information, aiming to enhance patient health and healthcare delivery, for example, via wearables, the internet, mobile applications, or text messaging [20, 21]. By overcoming challenges such as geographic isolation, transportation difficulties, limited appointment availability, and high healthcare costs, DHIs expand access to underserved populations [22–24]. Moreover, the technological flexibility of DHIs allows for tailored adaptations to meet the cultural, linguistic, and contextual needs of diverse populations [25]. To this end, DHIs can enhance treatment outcomes and sustain engagement [26, 27]. Recent innovations in generative artificial intelligence (e.g., large language models) [28–30], further enable real-time personalization and dynamic adaptation of interventions, paving the way for tailored solutions at scale. Simultaneously, regulatory bodies such as the EU AI act are introduced to ensure more equitable DHIs. Consequently, DHIs hold significant promise as tools to address health inequities and drive progress toward a more equitable healthcare landscape.

However, DHIs also risk exacerbating the digital divide, e.g., disadvantaging populations with limited access to technology [31] lower digital (health) literacy [32] lower socioeconomic status [33, 34] or underrepresented data in artificial intelligence (AI) models [35]. For example, a significant portion of the US population still lacks broadband access, with disparities more pronounced among low-income households, with 38% of households earning less than $20,000 lacking a broadband subscription [33]. Many low-income households share devices, limiting their ability to use DHIs [34, 36]. Older adults, ethnic minorities, and low-income individuals often have lower digital literacy, hindering their ability to engage with DHIs effectively [37–39]. These effects are also referred to as the *inverse care law*, which describes the phenomenon that individuals with more resources often possess better access, skills, and awareness of these interventions than those with fewer resources [40]. Furthermore, increased integration of AI raises additional challenges due to inherent bias. AI models often learn from historical data that may reflect existing health disparities [35, 41] which can perpetuate inequities if the data is biased against certain groups [42–44]. Biases can also arise during the design and development of AI algorithms, leading to unfair outcomes for priority populations [45–47]. Even if an AI model is unbiased, how clinicians and patients use it can introduce bias, affecting the outcomes [45]. Thus, as healthcare systems and healthcare delivery become increasingly digitalized, it becomes imperative to prioritize health equity as a fundamental objective and to reframe approaches to design, evaluate, implement, and scale DHIs [24, 48–52]. 

Despite broad consensus regarding the need for equity-centered design, there remains a critical gap in the form of actionable guidance for both research and practice. While there are frameworks introducing equity in DHIs (e.g., human-centered design [53] or community-based participatory research approaches [53]), these often either lack specificity or offer too much flexibility that designers and developers are left with limited guidance for consistent implementation [20, 54, 55]. Design principles are foundational guidelines that codify and formalize design knowledge in an accessible form [56] and are typically derived from extensive experience and/or empirical evidence [57]. They have become the predominant way to specify design knowledge [58] and have shown to be effective across various domains, e.g., improved user experience in human-computer interaction [59], more equitable education approaches [60], higher engagement in low-literacy individuals with e-health [61]. As such, design principles are a promising avenue to bridge this gap between knowledge and action by distilling equity considerations into actionable guidance tailored to DHI development.

Thus, we aim to develop *equity by design* principles that center health equity in DHIs and answer the following research question: **What design principles promote health equity** in DHIs?

## Methods

**Fig. 1:**
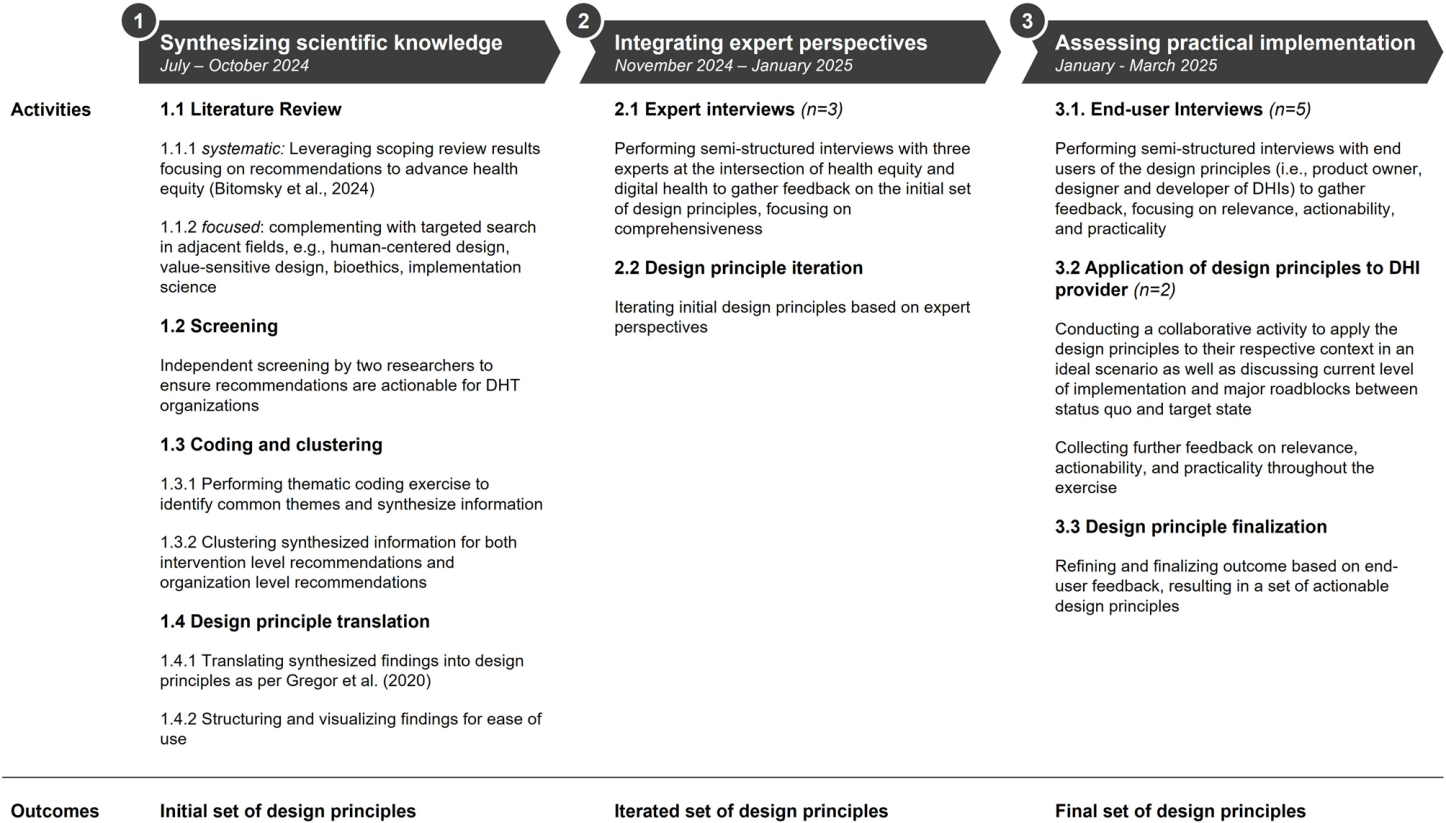
Overview of methodological approach

We followed three steps to answer our research question and develop a set of actionable, evidence-based design principles for promoting health equity in DHIs. Figure 1 provides an overview of the three steps. In the first step, we synthesized existing scientific knowledge. We then incorporated feedback from three experts in Step 2. Finally, we challenged the resulting design principles through end-user workshops with five practitioners to ensure their relevance (Step 3.1) and applicability (Step 3.2).

### Step 1: synthesis of scientific knowledge

To synthesize existing scientific knowledge, we employed two steps. First, we leveraged the findings from a scoping review focusing on frameworks and guidelines on advancing health equity [62]. The scoping review encompasses 38 studies from 2010 to 2023 and extracted 559 recommendations, which were synthesized into 82 recommendations across the dimensions *policy and government* (*n* = 27), *organizations and systems* (*n* = 31), *community* (*n* = 4), *individual* (*n* = 3), *and intervention* (*n* = 17). Most of the included works did not have a specific population focus (76%), though some studies specifically addressed people of color, rural populations, women, or low-SES groups. For the scope of this work, we included all studies that put forward recommendations on the organizations and systems level as well as on the intervention level, as these are within the sphere of influence for organizations designing and developing DHIs. Second, to complement the studies identified from the scoping review, we performed pragmatic, targeted searches in adjacent fields such as human-centered design, value-sensitive design, bioethics, and implementation science. Searches were initially performed across PubMed, Scopus, and Web of Science and focused on the following key words: (inequit* OR equit* OR human-centered OR customer-centered OR personalized OR value-sensitive OR ethic*) AND (health* OR design OR implementation OR evaluation OR health technolog* OR health intervention*). Relevant studies were then further used for forward and backward searches.

The collected data was then screened to ensure that the recommendations are actionable for DHI organizations, i.e., within their immediate sphere of influence. Screening was performed independently by two researchers to minimize bias. To analyze the data, thematic coding was performed to identify recurring themes and synthesize information. The synthesized data was then clustered into distinctive groups to reduce complexity and improve readability: for the recommendations on the DHI level, we adopted the structure put forward by the scoping review, i.e., needs assessment, design and development, implementation, and evaluation and dissemination [62]. For the recommendations on the organizational level, four overarching cluster emerged from the literature: *strategy*,* people*,* processes and structure*, and *partnerships and advocacy*.

Finally, the resulting synthesis was translated into an initial set of design principles using the structure proposed by Gregor et al. (2020): aim *(why)*, implementer *(who)*, context *(when)*, mechanism and sub-mechanism for more detail *(how)*, and rationale *(because)* [56]. They further propose to include a design principle name or title to enhance memorability. For ease of use, the design principles were structured into a coherent visual representation, featuring an inner layer focusing on the DHI, and an outer layer depicting the organizational context in which the DHIs are developed. Furthermore, we grouped all design principles that reference involving communities and other stakeholders for co-creation, participatory approaches etc. in another inner layer.

### Step 2: expert interviews

The second step involved refining the initial set of design principles through semi-structured expert interviews, focusing on the overall comprehensiveness of the principles. Three renowned experts at the intersection of health equity and digital health were interviewed, all with 10 + years of experience in their respective fields. All experts received an interview guide and the initial set of design principles (incl. all details on sub-levels) a minimum of seven days before the interview for preparation. Each interview was conducted online and lasted approximately 60 min, of which approximately 50 min were dedicated to substantive feedback across three overarching themes: *comprehensiveness and relevance of the principles*, their *feasibility and implementation potential*, and suggestions for *refinement and improvement* (see supplementary material SM.1 for interview guide). The interviews were recorded and transcribed, and the collected feedback was incorporated into revisions of the design principles between each interview.

### Step 3: End-user workshops

In the final step, we conducted end-user workshops to (a) challenge the theoretically derived principles from a practical perspective *(end-user interviews)* and (b) to think through the real-world application of the principles *(application of design principles)*.

For this step, we focused on the German healthcare context, which, despite offering universal healthcare coverage, exhibits notable health disparities across various population groups. For instance, individuals with a migration background [63] those with lower socioeconomic status [64] and people living in rural areas [65] often experience higher rates of unmet healthcare needs and worse health outcomes. This is further amplified in case of intersectional factors (e.g., being an immigrant woman) [66]. These structural inequities underscore the need to center equity in the design of digital health interventions.

Simultaneously, Germany was one of the first to pass their Digital Healthcare Act, which allows for reimbursement of digital health interventions, also called DiGAs, by the statutory health insurance [67]. Thus, the specific requirements defined by the Federal Institute for Drugs and Medical Devices provides a clear context and allows for comparability. Further, as of 2023, all permanently listed DiGAs have provided a positive care effect in the form of a medical benefit – instead of e.g., structural and procedural improvements –, thus meeting higher standards than required by the DiGA guideline [68]. While the DiGA framework provides a highly standardized regulatory environment, it does not mandate equity-specific requirements. This creates a compelling case for investigating how equity by design principles can be meaningfully applied within such a context.

We collaborated with two DiGA providers: *DiGA1* was founded 2016 and has four permanently listed DiGAs, focusing on mental health. Their first DiGA was accredited in 2020, and they have since treated around 40.000 patients. *DiGA2* was founded 2015 and was accredited in 2020. Since then, they have treated more than 170.000 patients with their DiGA offering therapeutic training for acute and chronic back pain, ranking them the most prescribed DiGA in Germany in 2022. Both conditions, mental health and chronic back pain, disproportionately affect marginalized populations: For instance, LGBTQIA + individuals face over twice the likelihood of experiencing a common mental disorder within their lifetime [69] and lower-income individuals face similar risks compared to their higher income counterparts [70]. Similarly, prevalence and intensity of back pain have been associated with lower economic status and education [71, 72] and are higher amongst people living in rural areas [73]. As such, centering health equity in the design and development of DHIs is of high importance.

The workshops were conducted online and lasted 90 (*DiGA1*) and 120 (*DiGA2*) minutes, respectively. From *DiGA1*, the product owner and head of market access, both with 5 + years of experience in digital health, participated in the workshop. From *DiGA2*, the product owner (9 + years), a UI/UX designer (5 + years), and the chief technology and product officer (10 + years) participated in the workshop. All participants received a full workshop guide and the iterated set of design principles (incl. all details on sub-levels) a minimum of seven days before the interview for preparation.

#### End-user interviews

In the first phase of the workshop, the design principles were presented, and the participants had the chance to provide first reactions and structured feedback for approximately 30 min. The objective was to challenge the theoretically derived principles from a practical perspective to gain deeper insights into their impact potential. Thus, participants were guided through semi-structured interviews along three dimensions: *relevance and usefulness*, *understandability and actionability*, and *practicality and implementation* (see SM.2 for interview guide).

#### Application of design principles

This initial feedback was followed by a collaborative activity *(60–90 min)* to think through how the design principles could be applied in their respective organizations in a target state. To minimize time commitment from the participants, *Author 1* prepared an initial draft of the applied design principles based on publicly available information from each organization, e.g., company website, reports, research, news articles. This draft was used as the basis for a rich discussion, during which participants were encouraged to think aloud, challenge assumptions, and collaboratively develop their exemplary target state application. Throughout this activity, the current level of implementation and major roadblocks between status quo and target state were continuously discussed. In addition to the feedback collected throughout, participants had the opportunity to provide final feedback on relevance, actionability, and practicality of the design principles at the end of the workshop. The workshops were recorded and transcribed.

Finally, the collected feedback was coded and thematic analysis performed to identify overarching areas of improvement. The feedback was incorporated to derive the final set of design principles presented in this study.

## Results

To address our research question, we developed a set of 25 design principles for centering health equity in DHIs. The supplementary material SM.3 visualizes the evolution of the design principles throughout the process.

### Literature synthesis

In the initial literature synthesis, we included 43 studies of which 31 were identified directly from the scoping review [62] and 12 were added through the targeted search. They span across health sciences, social sciences and humanities, life sciences, and physical sciences, based on Scopus’ *All Science Journal Classification Codes (ASJC).* Specifically, 78% stemmed from health sciences (*n* = 28), 8% from both social studies and humanities (*n* = 3) and physical studies (*n* = 3), and the rest from life sciences (*n* = 2). More than three-quarters of the included studies (*n* = 28) were published in the past 5 years, with the oldest study from 2012. An overview of all included studies and their key characteristics (e.g. publication year, Scopus ASJC categories) can be found in the supplementary materials (SM.4).

The analysis of these studies resulted in an initial set of 26 design principles, of which 15 were directly related to the intervention, while 11 focused on the organizational context in which DHIs are developed (cf. SM.3).

### Interview synthesis

During the interviews, all interviewees (in the following referred to as E1, E2, E3) underlined the comprehensiveness and relevance of the presented principles and acknowledged the overall methodological approach. The value of including organizational context principles was especially highlighted given the limited guidance for private sector organizations.

*“Some of these aspects are less discussed in the literature than others*,* especially when it comes to how the private sector should embed certain principles from the onset. As of today*,* there is often not more than quite aspirational claims about this*,* so it’s so important to include this here.”* [E2].

When it comes to uptake of health equity principles beyond aspirational claims, aligning ethical imperatives with strategic incentives may strengthen implementation. One expert noted the potential of equity as a “competitive edge” [E3]. As such, equity should not be treated merely as a compliance issue or an afterthought but as a potential differentiator for companies. By focusing on equity from the outset and positioning equity as part of their strategy and identity, startups can attract smart capital. This could ultimately lead to better products and ethically driven competition. This is not to reduce the normative importance of health equity, but to highlight that investor interest and brand differentiation could help reinforce equity’s relevance in real-world business decisions and thus drive adoption.

*“Embedding health equity into your overall strategy can be such a strong multiplier. If you’re a startup*,* you have to attract investment. You can make a difference by attracting smart capital*,* not just any capital*,* by making equity part of your signature approach to innovation. And I hope to see competition in the marketplace of startups and companies*,* but competition that is ethically driven*,* not just about who gets the most funding or who launches first.”* [E3].

However, they pointed out that organizations are only “*a puzzle piece on the way to health equity*” [E1]. As such, they are not solely responsible for achieving global health equity but have a “*great responsibility to make an important contribution alongside regulation and politics*” [E1].

Suggestions for refinement were fourfold and encompassed (1) including the *concept of epistemic injustice*, (2) recognizing the *context of implementation*, (3) accounting for *tradeoffs*, and finally, (4) *wording and streamlining* suggestions:

#### Epistemic injustice

The interviews underscored the critical issue of epistemic injustice in community involvement. Epistemic injustice refers to the unfair treatment of individuals in their “capacity as knowers”, often manifesting as testimonial injustice — where a person’s credibility is unjustly deflated due to prejudice — and hermeneutical injustice — where there is a lack of shared interpretive resources, disadvantaging certain individuals in making sense of their experiences [74]. As a result, some voices are heard more than others and some people are better able to articulate or understand their needs than others, creating a “*significant blind spot*” [E1]. This gap is exacerbated when digital health applications are designed primarily with a business case in mind rather than as social innovations.

*“When an app is conceived as a business case rather than a social innovation*,* this can already create a fundamental equity gap– it is known that many people have certain needs*,* but they are not addressed because they do not represent an obvious business case. "* [E1].

To account for this feedback, we included the concept of epistemic injustice in the community involvement box spanning around all principles based on this approach.

#### Context of implementation

One of the experts (E2) highlighted two primary sources of inequity in AI-based systems, data representativity and system behavior and context. They emphasize that the principles should account for the specific contexts in which DHIs are implemented, such as infrastructure, socioeconomic factors, and contextual adaptability.

*“Sophisticated systems might work well in certain contexts or for certain conditions*,* depending on the availability of robust infrastructure and the capacity to pay. But they might not work equally well in other contexts*,* putting low- and middle-income countries — or even socioeconomically deprived areas within the same country — at a disadvantage.”* [E2].

*“Organizations must consider if the use of DHIs is a privilege or a necessity for people with fewer resources. As such the question whether a digital health solution closes a gap or widens it largely depends on which alternatives it replaces and the context in which it’s deployed.”* [E3].

We have integrated this feedback by extending an existing principle on implementation context to include broader context considerations.

#### Tradeoffs

Tradeoffs highlight the need for prioritization within the design principles, e.g., maximizing technical accessibility might not always be possible while simultaneously enabling maximum financial accessibility. As such, we have included arrows between the inner DHI layer and the outer organizational context to visualize the interaction and interdependencies between these layers.

*“When it comes to equity*,* the question arises: Who can afford what? Someone with little money might choose the free app without fully understanding what happens to their data — while someone with more resources can afford the paid*,* privacy-friendly alternative. Data protection is often seen as a universal requirement*,* but in practice*,* there is always room for flexibility. Some groups are more likely to be forced into accepting lower data protection standards.”* [E1].

*“Organizations who use off-the-shelf solutions are basically using a system that is efficient*,* but those who can afford to customize it to their specific needs are at a further advantage — beyond just being richer to begin with — because they are going to have a better version of the same model.”* [E3].

#### Wording and streamlining

Finally, we received feedback on the overall wording and streamlining of the design principles. One expert suggested merging two principles focusing on building and sustaining organizational capacity for health equity action as they are closely linked. They further suggested using the terms “*monitoring and oversight*” instead of “*follow-up*” [E2] during the evaluation and dissemination phase, given “*once a system is in clinical use*,* monitoring is crucial to identify biases that may not be predicted during design and development stages*” [E2]. They further recommended avoiding the term customer as it sounds “*too business-like in the context of digital health*” [E2] and instead focusing on end-users or target populations. Another expert suggested extending a principle on privacy concerns by data protection, as “*privacy is often equated with data protection*” [E1]. Finally, an expert recommended reviewing the language of the principles to make sure that the “*link to equity is clear and direct*” [E3], and, if necessary, adjusting the phrasing to make the focus on equity more apparent.

All wording and streamlining suggestions were implemented across the principles. This resulted in a revised set of 25 design principles, with 15 directly related to intervention and 10 associated with the organizational context (cf. SM.3).

### End-user workshop synthesis

While the expert interviews contributed the concepts epistemic injustice, implementation context, trade-offs, and language improvements, the end-user workshops focused on the revised framework as a whole, exploring how the Full set of 25 principles could be applied in practice. During the end-user workshops, all participants (referenced as D1.1, D1.2, D2.1, D2.2, D2.3) underlined the importance and relevance of the presented principles and acknowledged that if applied effectively, they would lead to more equitable DHIs.

*“I strongly believe these could create a positive impact if applied effectively.”* [D1.1].

*“Security used to be an afterthought*,* but now it’s built into the process— this needs to happen for equity too. It can’t be enforced from above; it must be integrated from the start. So*,* I really like this approach.”* [D2.1].

Several participants appreciated the structured approach of extending the principles by organizational context, stating that “*it’s really helpful and quite cool to think beyond product-level principles*” [D2.2]. However, one participant pointed out that the relevance and impact of specific principles may vary depending on regulatory and market contexts. For instance, in highly regulated environments like the German DiGA market, flexibility in applying these principles is limited, making certain equity-focused approaches more challenging to implement.

*“German DiGAs are heavily regulated*,* limiting differentiating options. If you have more flexibility in payment models that allow you to upgrade certain features*,* for example*,* this could create a competitive edge and set other incentives to apply these.”* [D1.2].

All participants confirmed that the principles provide overall clear guidance and inspire actionable steps, underlining their overall understandability and actionability. However, some principles required further clarification to ensure actionable implementation. The wording was revised in the session and the feedback directly implemented.

*“Most of the principles are clear*,* but some need more explanation. For example*,* ‘communicate transparently’—I’m not sure what that means in a practical sense and who the target audience of this communication would be. Or ‘build community capacity’*,* this could be understood from multiple perspectives. I think this needs a bit more clarification.”* [D1.1].

*“I would not speak of ‘maximizing financial accessibility’ – it sounds too much business-like and maximizing profits. I would opt for something like ‘enabling’ or ‘allow for’.”* [D2.1].

During the collaborative activity, the design principles were exemplary applied, and the current level of implementation was discussed. To set contextual boundaries of the application, we agreed upon a fitting scenario, i.e., developing a new anxiety therapy module *(DiGA1)* and developing a therapy module for knee osteoarthritis *(DiGA2)*. While the principles were overall considered compelling in theory, all participants pointed out challenges hindering successful application. The challenges can be summarized into three overarching topics: (1) practical implementation, (2) structural and regulatory barriers, and (3) resource constraints.

#### Practical implementation

Principles related to user engagement and personalization can be difficult for organizations to implement. Access to end-users was considered as “*already hard*” [D2.3] and while a more diverse sample would be great, it would be “*difficult to manage in practice*” [D2.2]. Another discussed challenge concerned sustaining user engagement with culturally tailored strategies. This topic was perceived as “*heavily researched*” [D1.2] but “*just not feasible as of now*” [D1.1]. Another raised critical challenge related to the role of healthcare providers in digital health adoption. Participants emphasized that even the most well-designed, equitable products will struggle to gain traction if prescribing doctors are not adequately engaged. This underscores the need for stronger integration between DHIs and clinical workflows.

*“The relationship between the user and their prescribing doctor is critical. If doctors aren’t on board with the product or don’t understand its value*,* that’s going to affect how and if the patients use it. As such*,* healthcare providers are a huge lever in DHI adoption.”* [D1.2].

#### Structural and regulatory barriers

Participants pointed to broader systemic barriers that make it difficult for digital health companies to integrate equity into their products and workflows. Regulatory restrictions in Germany were frequently cited as a major obstacle to continuous innovation.

*“Once we complete our required trials*,* we can’t adapt the program without redoing them. This makes it hard to stay up to date with new scientific evidence. As such*,* regulation in Germany actually hinders innovation. It prevents us from continuously integrating the latest research.”* [D2.2].

*“We’d like to collect more diverse data*,* but it’s simply not allowed.”* [D2.3].

Another key structural challenge is the fragmentation of the digital health ecosystem. Many healthcare providers struggle with integrating multiple digital solutions into their existing workflows, making widespread adoption more difficult.

*“System integration is such a challenge due to fragmentation. A physician managing five different apps from five different DiGAs? That’s unrealistic. A more streamlined approach is needed.”* [D2.1].

#### Resource constraints

A recurring theme in the discussions was the tension between regulatory demands, business sustainability, and the capacity to innovate. Many organizations struggle to allocate resources toward equity efforts when they are already stretched thin by compliance requirements and market pressures.

*“One major challenge in our market is competing demands—tons of regulatory requirements that are extremely time-consuming and complex*,* often with very tight deadlines. At the same time*,* we’d love to work on product features that actually benefit users or drive growth*,* but there’s just never enough capacity.”* [D1.1].

Limited financial and human resources further exacerbate these constraints. Startups in particular often lack the budget to hire dedicated teams for health equity initiatives, making it difficult to implement these principles in a meaningful way.

*“Some of our biggest constraints are money and time. We do not have the resources to do everything simultaneously and then consequently*,* this would lead to longer time-to-markets which we often just cannot afford.”* [D1.2].

*“Beyond budget constraints we also don’t have the personnel with relevant equity expertise and experience. It’s already challenging enough to get talents*,* but chances of finding the right profiles that also come with the knowledge to advance health equity are probably close to zero.”* [D2.2].

Some participants contrasted their experiences in well-funded versus resource-constrained organizations, highlighting how financial backing can dramatically influence product development approaches. Organizations with larger budgets have the luxury of conducting extensive user research, iterating on concepts, and investing in long-term equity strategies—which is rarely feasible for smaller companies.

*“At my previous company*,* which had a huge budget*,* product development was completely different. We could spend months on user research*,* conduct unlimited interviews*,* and develop concepts with the necessary personnel. That really shows how much of a factor money is.”* [D2.3]. Given these challenges and constraints, prioritization becomes essential. Participants noted that companies must constantly evaluate the trade-offs between effort and impact, making difficult decisions about which initiatives to pursue. Without a structured way to prioritize efforts, organizations may become overwhelmed and ultimately discouraged from engaging with equity initiatives.

*“In reality*,* it can be quite disappointing to see that developing the perfect product isn’t the only factor*,* there’s also the need to keep the business running. If we were to apply all of this realistically in practice*,* I think it would be very difficult for everyone. A prioritization approach would be extremely helpful*,* so organizations don’t get overwhelmed and with that discouraged.”* [D1.1].

*“We need some kind of prioritization*,* like if a company does ‘XYZ’ [! sic]*,* are they already on the right path. A step-by-step approach would be super helpful.”* [D2.1].

Despite the challenges highlighted by the participants, they encouraged us to keep the principles “*ambitious and aspirational*” [D1.2] to act as a “*north star*” [P5]. Rather than omitting principles, they underlined the value of maintaining a version that “*pushes boundaries and challenges organizations to think beyond current constraints*” [D2.2]. A full overview of the applied principles for both DiGAs and their current levels of implementation, including the respective roadblocks, can be found in SM.5.

Eventually, integrating the feedback from the end-user workshops (cf. SM.3) resulted in the final set of 25 design principles, with 15 directly related to the DHI and 10 associated with the organizational context, which will be introduced in detail in the following section.

### Final set of equity by design principles

**Fig. 2:**
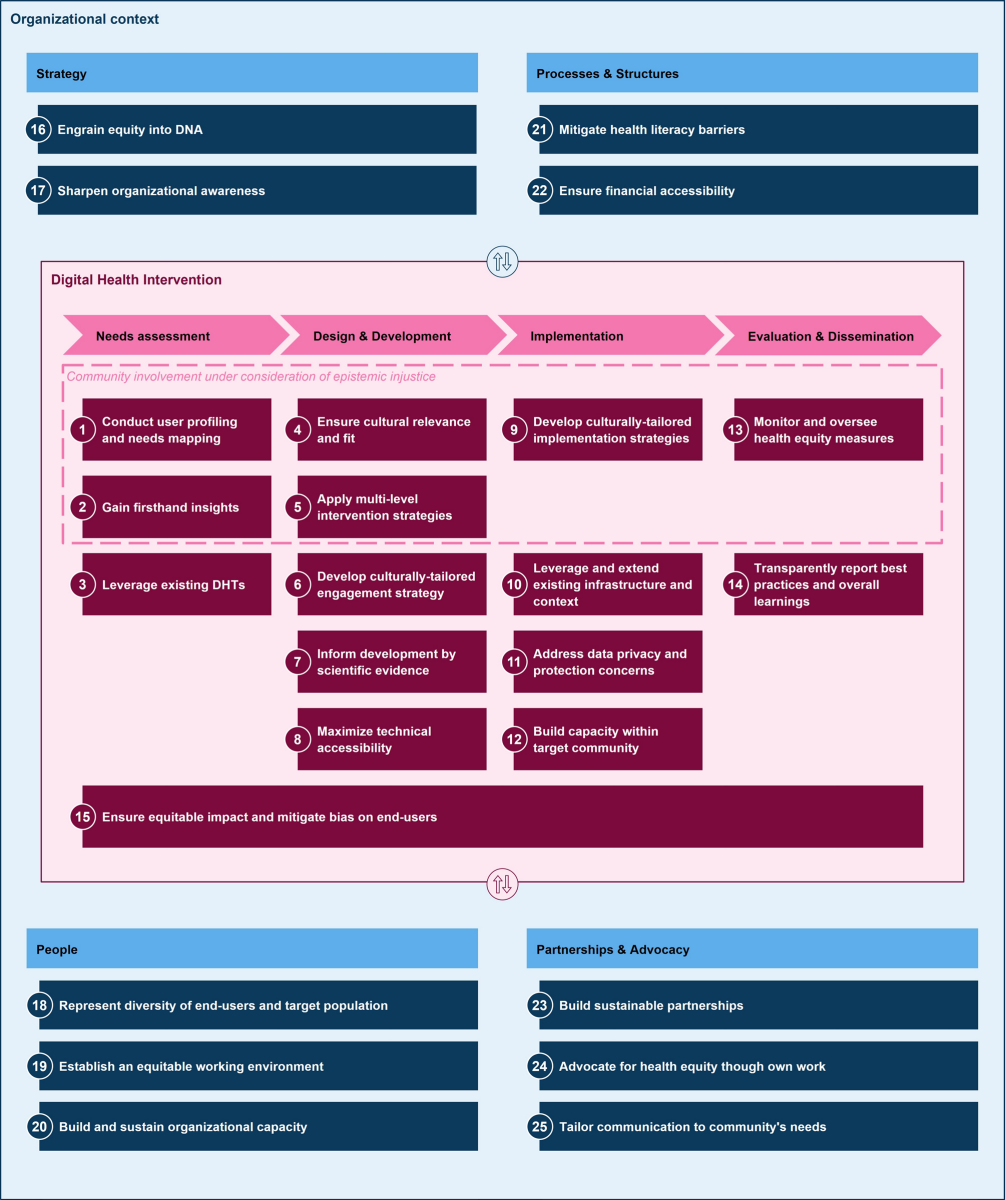
Final set of equity by design principles to center health equity in digital health interventions. Note: This figure consists of two layers: the digital health intervention layer (inner box in magenta) and the organizational context (outer box in blue). The digital health intervention layer follows four process steps whereas the organizational context consists of four foundational components. Each numbered element represents a specific equity by design principle. One principle (15) thereby spans across all four phases of the process. Another common element throughout the process is community involvement under consideration of epistemic injustice, which is indicated by a dotted line. Further, there are inherent trade-offs and interdependencies between the layers, which are indicated by the two-way arrows at the top and bottom of the digital health intervention layer

The final set of equity by design principles is depicted in Fig. 2 and further described in detail in SM.6. In SM.6, each design principle is outlined following the same structure as recommend by Gregor et al. [56]: design principle name (seen in Fig. 2), purpose, implementor, context, mechanism, sub-mechanism, rationale, and supporting sources (cf. supplementary materials SM.6).

#### Digital health intervention

At the DHI layer, design principles are clustered along the process steps *needs assessment* (*n* = 4), *design & development* (*n* = 5), *implementation* (*n* = 4), and *evaluation & dissemination* (*n* = 2).

- During *needs assessment*, the focus lies on developing tailored strategies for inclusive intervention design for identified user profiles (e.g., [52, 75–77]), gaining firsthand and in-depth insights into disparities, root causes and contextual realities (e.g., [78–80]), to build upon existing, relevant DHIs (e.g., [81–83]), and to ensure equitable access and impact on all end-users (e.g., [75, 76, 79, 84]).
- During *design and development*, design principles center around cultural relevance and fit by utilizing participatory approaches throughout (e.g., [49, 82, 85]), targeting various levels of influence from the patient to the microsystem and organizations [86, 87], tailoring content and behavior change mechanisms to user characteristics to sustain engagement (e.g., [49, 76, 83, 84, 88–90]), building upon findings from evidence-based interventions and locally relevant programs [78, 85], and maximizing technical accessibility by ensuring interventions are agnostic to devices, operating systems, mindful of Wi-Fi and cellular data availability etc. (e.g., [50, 76, 89]).
- During *implementation*, design principles focus on culturally tailored implementation strategies (e.g., [52, 82, 88]), acknowledging and extending available infrastructure and context of implementation, including both technical as well as social contexts (e.g., [75, 78, 89]), addressing data privacy and data protection concerns, focusing on concerns relevant to the target group, e.g., based on historical discrimination and stigma [75, 89], and finally building community capacity by hiring and training community members, providing necessary resources, support and establishing a feedback system [78].
- Finally, during the *evaluation and dissemination*, design principles center around following through on health equity promises with stringent monitoring and feedback loops (e.g., [79, 84, 91]) and transparent communication of best practices and learnings from unintended consequences [75, 76].

Across all steps of the process, principles building upon the concept of community involvement can be found. An inner layer encompassing all relevant principles highlights the central element of considering epistemic injustice throughout these co-creation activities. 

#### Organizational context

To address the organizational context in which DHIs are designed and developed, design principles are clustered along *strategy* (*n* = 2), *people* (*n* = 3), *processes & structures* (*n* = 2), and *partnerships & advocacy* (*n* = 3).

- *Organizational strategy* focuses on making health equity a strategic priority and committing to clear health equity goals (e.g., [87, 92–96]). It also enhances organizational awareness by understanding workforce compositions and decision-making processes [92].
- *People* centers around attracting and engaging talent from marginalized populations to represent the diversity of your end-users and target populations (e.g., [77, 80, 81]), creating an equitable work environment by challenging assumptions, adopting a zero-tolerance culture towards racism, addressing power imbalances, etc. (e.g., [93, 94, 97]), as well as building and sustaining organizational capacity for health equity actions through regular mandatory training, bi-directional learning, and effective resource allocation (e.g., [95, 98, 99]).
- *Processes and structures* refer to developing and providing necessary health literacy support to end-users to mitigate barriers (e.g., [77, 80, 100]) and maximize financial accessibility through sustainable funding options, accreditation systems, and/or public safety net settings (e.g., [49, 77, 87, 97]).
- Finally, *partnerships and advocacy* refer to building sustainable community partnerships based on shared goals, trust, and mutual respect (e.g., [77, 83, 87, 93]), engaging with local boards, community groups, and political representatives to create visibility for own health equity efforts and share learnings [80] and last but not least prioritizing equity initiatives in communication strategy and tailoring overall communication to target population’s needs [81, 91, 92].

Furthermore, there are certain interdependencies and tradeoffs between the organizational context and the design and development process. For example, the partnerships an organization builds impact how easy it is to involve relevant community members throughout development. Depending on the country an organization operates in, different funding sources are available that impact the DHIs’ financial accessibility, impacting the number of technical features and tailored intervention components that can be included. Organizations wanting to optimize for health equity impact need to consider these interdependencies.

## Discussion

This study aimed to develop design principles to center health equity in DHIs. The research highlights the importance of promoting health equity beyond the DHI level across the organizational context, addressing systemic barriers contributing to disparities in health outcomes. By synthesizing insights from literature, expert interviews, and workshops with German digital health companies (DiGAs), we proposed 25 actionable design principles that organizations can adopt to embed equity into their practices. Several key themes emerged from our research.

A critical insight from our study is that product-level design decisions alone are not enough to achieve equity; the organizational context that shapes digital health innovation plays an equally important role. Design principles on the organizational level refer to structural and strategic factors – such as overall strategy, governance models, partnerships, funding mechanisms, and regulatory engagement — that influence an organization’s capacity to implement equitable design practices. These differ from intervention-level design principles, which focus on the direct development of a technology (e.g., user-centered design, accessibility, and participatory approaches). Our findings indicate that certain organizational choices can facilitate or constrain the implementation of equity principles. For instance, the nature of an organization’s partnerships can affect the extent of community involvement in co-design efforts. At the same time, business models influence whether financial accessibility can be prioritized over the delivery of highly personalized, technology-driven care. These organizational factors introduce inherent trade-offs – such as balancing financial sustainability with equitable access. Notably, our analysis of two German DiGAs suggests the organizational-level principles remain largely unaddressed. This underscores a common challenge: equity considerations are often treated as product features rather than structural commitments embedded in an organization’s mission and operations. However, if these broader structural determinants are overlooked, even well-intentioned design efforts may fall short of their intended equity impact. Future research should explore how alternative business models could balance financial sustainability with equity goals. Approaches such as tiered pricing, cross-subsidization, or strategic partnerships with public health entities may offer viable pathways for sustaining equity-driven DHIs while ensuring long-term financial viability.

Another key challenge identified in this research is the practical feasibility of implementing all 25 equity by design principles. Given constraints in time, funding, and expertise — particularly for smaller startups — organizations require structured prioritization strategies to phase in equity principles in a meaningful and scalable manner. Prioritization frameworks and tools could support this process. For instance, an impact-feasibility matrix could help organizations focus on high-impact, low-cost principles in early development stages while gradually integrating more resource-intensive practices. A phased implementation model could guide companies through progressive stages of equity integration, from foundational practices (e.g., inclusive language and accessibility standards) to advanced strategies (e.g., embedded community governance structures). Developing customizable self-assessment tools that help organizations evaluate their current adherence and identify priority areas could further support companies navigating equity implementation in practice.

The final insight from this research we want to highlight is the critical role of epistemic injustice in health equity initiatives, i.e., the concept that certain marginalized groups have fewer resources, confidence, or opportunities to advocate effectively for their needs than others. As many equity-focused principles rely on active community involvement, it is vital to consider how to mitigate the impacts of epistemic injustice so as not to leave behind those already marginalized inadvertently. This underscores the need for equity strategies that actively mitigate power imbalances during needs assessments and co-design processes, ensuring that all voices are genuinely heard and represented regardless of social capital or advocacy skills. Additionally, this points to the inherent tension between equitable DHIs as a business case versus a social case. Business sustainability often depends on targeting large, financially viable user groups, which may inadvertently exclude marginalized populations. This raises critical questions about how to align equity goals with business imperatives, particularly in the context of for-profit DHI development. While the workshops with DiGA providers did not explicitly focus on epistemic injustice, participants repeatedly pointed to challenges such as limited end-user engagement, lack of equity expertise, and resource constraints that reflect some of its real-world implications. These findings highlight the importance of designing equity strategies that account for both structural constraints and power imbalances in practice.

While this work contributes to advancing health equity by providing actionable guidance to practitioners and policymakers alike, it is not without limitations. One notable constraint is the exclusive focus on the German DiGA context. Germany’s regulated DiGA framework provides an innovative and structured pathway for digital therapeutics, ensuring that products meet standards for clinical effectiveness, data security, and reimbursement eligibility. However, this framework also imposes constraints: regulatory requirements may harmonize aspects of DHI development, making it difficult to distinguish between genuine equity-driven efforts and compliance-driven adaptations. Furthermore, it may hinder implementation of necessary equity-driven measures such as data-driven decision-making given strict data protection and privacy regulations. Thus, the findings may not be directly generalizable to other markets. Future research should explore how these principles can be applied in diverse geographic and regulatory contexts, where regulatory frameworks and resource constraints differ significantly. Moreover, with the focus on German DiGAs we solely investigate digital therapeutics as a subgroup of DHIs. Future research should examine how these equity-focused principles can be applied to other DHIs, e.g., preventive health technologies. Given the growing importance of prevention in public health, this is a critical area for further investigation.

A related challenge is how to create sustained commitment to health equity across the DHI sector. While many organizations recognize the importance of equity, implementing these principles often competes with other operational priorities, such as achieving profitability, scaling market reach, or securing investor funding. Regulatory bodies could incentivize the adoption of equity principles by integrating them into accreditation frameworks, such as requiring equity assessments as part of reimbursement eligibility criteria for DiGAs. Additionally, targeted funding mechanisms, such as equity-focused innovation grants, could lower the barriers for startups seeking to integrate equity from the outset. Future research could investigate the feasibility and impact of such strategies to create a supportive environment for equitable innovation. Further, a cost-benefit analysis of incorporating equity considerations could be performed to investigate inherent financial benefits in addressing health equity. Given the tremendous economic opportunity of closing the healthcare gap [8, 16] incorporating equity considerations could be seen as a strategic advantage for digital health companies. As such, health equity could be recognized as a key success factor – on par with other success factors recently identified [101].

Finally, while our research benefited from experts and end-users through workshops and interviews, we acknowledge limitations in both participant composition and depth. Since this study was designed as a literature-driven effort and expert insights served to validate and contextualize the principles derived rather than to generate them inductively, the number of participants was intentionally limited to support depth over breadth. Yet, these interactions reflect a specific subset of perspectives that may be skewed toward organizations and individuals already interested in equity and thus may not fully capture views from actors less engaged with or critical of equity-centered approaches. Notably, the organizations represented in our end-user workshops primarily focus on mental health and chronic back pain – conditions that disproportionately affect marginalized populations, but which may also orient participants toward condition-specific interpretations of equity. While their expertise enriched the refinement of the principles, it may also have influenced the emphasis placed on certain design elements over others. Thus, further work is needed to capture insights from a broader range of DHI developers, particularly those operating outside the DiGA framework. Additionally, the experts involved in refining the design principles come from diverse disciplinary backgrounds, including digital health ethics, algorithmic accountability, and user-centered design and implementation science. While this range of expertise enhances the relevance and robustness of the principles, it may also have introduced implicit biases based on the experts’ professional orientations and prior work. Future research may benefit from engaging additional perspectives, including those from public health practice and patient advocacy.

In conclusion, this study provides design principles to center health equity in DHIs. Its findings underscore the necessity of moving beyond intervention-level design considerations to address health inequities in digital health. Future work should focus on developing a health equity readiness index, strengthening policy incentives for equity-centered innovation, and evaluating long-term equity outcomes in real-world DHI deployments. By embedding equity as a core strategic priority rather than an afterthought, digital health organizations can contribute meaningfully to reducing health disparities and fostering more inclusive healthcare ecosystems. Beyond the positive societal impact, this will likely yield further economic benefits when user-centered reimbursement strategies such as value-based care and pricing are employed.

## Supplementary Information

Supplementary Material 1.

## Data Availability

The data supporting the results will be made available upon reasonable request to the corresponding author.

## Abbreviations

AI: Artificial Intelligence
DHI: Digital Health Intervention
