# Supplementary material for: Equity by design principles for digital health interventions
Source: Int J Equity Health. 2025 Oct 14;24:271. doi: 10.1186/s12939-025-02645-6 (PMC12522812; doi:10.1186/s12939-025-02645-6)
Supplement: Supplementary file 1 — Supplementary Material 1. [file 12939_2025_2645_MOESM1_ESM.docx]

**Equity by Design Principles for
Digital Health Interventions**

*Supplementary Materials (SM)*

SM.1: Expert Interviews – Interview Guide *(60 min)*

Comprehensiveness and Relevance of Design Principles

*Objective: Assess the overall relevance of the design principles and gather insights on potential gaps or redundancies.*

- Do you think these design principles **address the critical aspects** necessary for fostering health equity in DHTs?
- Are there **any major areas** of health equity or design **that you feel are missing**?
- Are there **principles that feel redundant or overly broad**?
- If you were to **prioritize these principles**, which ones would you consider **the most critical for achieving health equity** in DHTs, and why?

Feasibility and Implementation

*Objective: Explore the practical implications of applying these principles in real-world contexts*

- **How feasible** do you think these principles are **for implementation** by designers, developers, and organizations working on DHTs?
- **What challenges do you anticipate** organizations might face in adopting these principles?
- Do you know of any **real-world examples** where similar principles have been **successfully implemented?**

Refinement and Improvement

*Objective: Solicit specific suggestions to enhance the clarity, usability, and impact of the principles.*

- Are the principles **written in a way that is clear and actionable**? How could the **language or framing be improved**?
- How might we even better **incorporate user-centered or community-driven approaches** into these principles?
- Based on your expertise, is there **any emerging research, framework, or perspective that we should consider incorporating** into these principles?

Final

- Is there **anything else you’d like to share or feel we haven’t discussed** that’s relevant to this project?
- Do you have **any advice for ensuring these principles are as impactful and actionable as possible**?

SM.2 End-user Interviews – Interview Guide (30 – 45 min)

Relevance and Usefulness

*Objective: Assess the overall relevance of the design principles and gather insights on their perceived usefulness*

- Do you believe **these design principles would lead to more equitable DHTs** if applied effectively? Why or why not?
- Are there any **principles that feel especially relevant** – or irrelevant – for your work?
- Are there any **equity-related issues** you face that **these principles don’t seem to address**?

Understandability and Actionability

*Objective: Evaluate whether the principles provide clear guidance and inspire actionable steps*

- Are the design principles and their descriptions **clear and easy to understand**?
- Are these principles specific **enough for you to know what actions to take**?
- Do you feel **any principles, terms or concepts** are **too abstract or unclear?**
- What **additional information, tools, or examples** would make these principles **easier to follow**?

Practicality & Implementation

*Objective: Uncover potential barriers or constraints to applying the principles in practice.*

- Are there **any principles you think would be difficult to implement** in your organization? Why?
- **What specific challenges** – such as time, budget, or resources – **might prevent you** from applying these principles?
- Do any principles **require significant organizational changes** to implement? If so, which ones?
- **What would make these principles easier** to implement in your context?

Final

- Do you think these principles **would benefit from any additional themes or focus areas**?
- What would make these principles **more valuable to you and your organization**?
- Is there **anything else you’d like to share or feel we haven’t discussed**?

SM.3 Evolution of the Design Principles

3.1 Initial set of design principles after literature synthesis (Step 1)


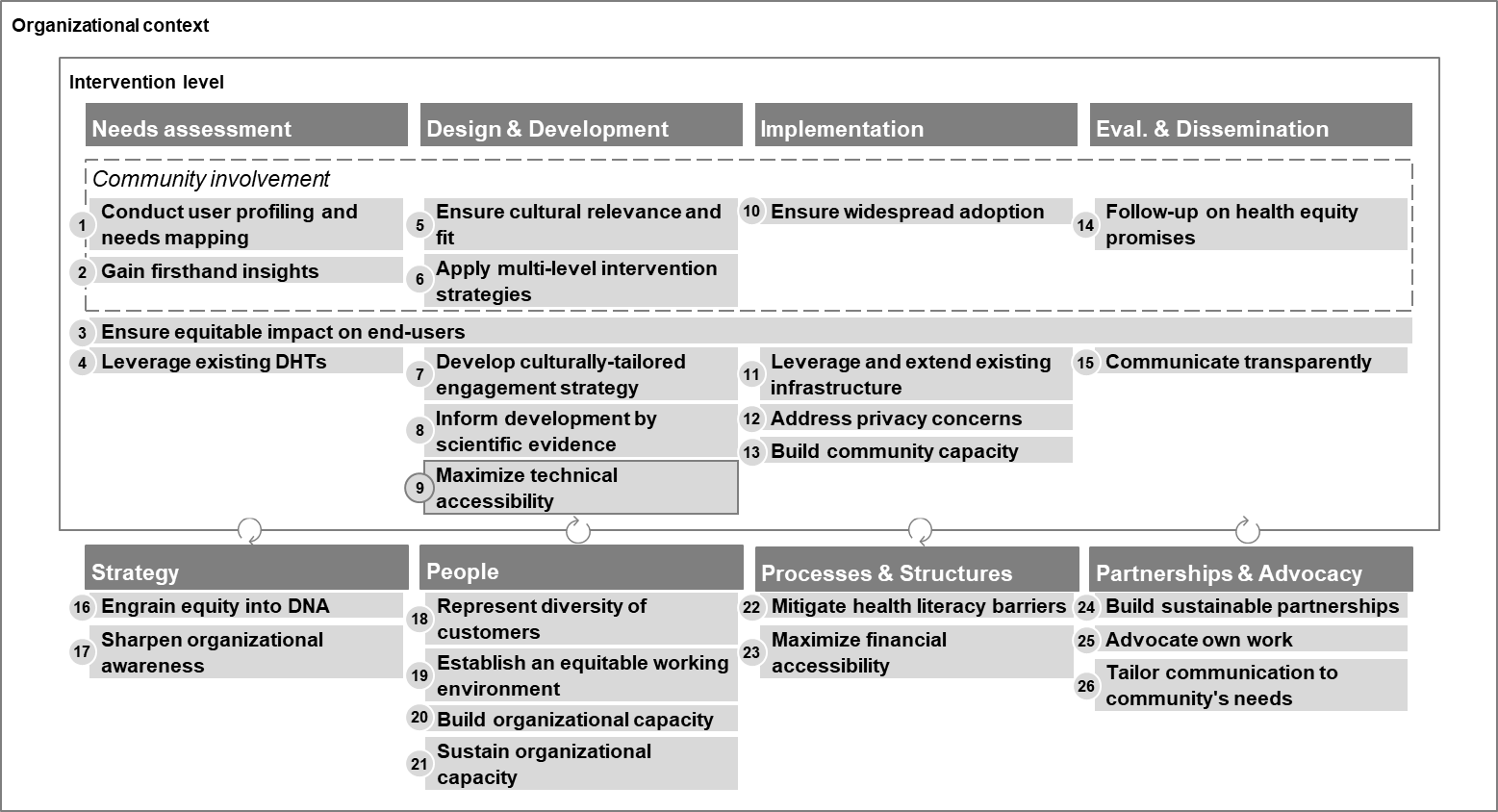


3.2 Design Principles after the expert interviews (Step 2)


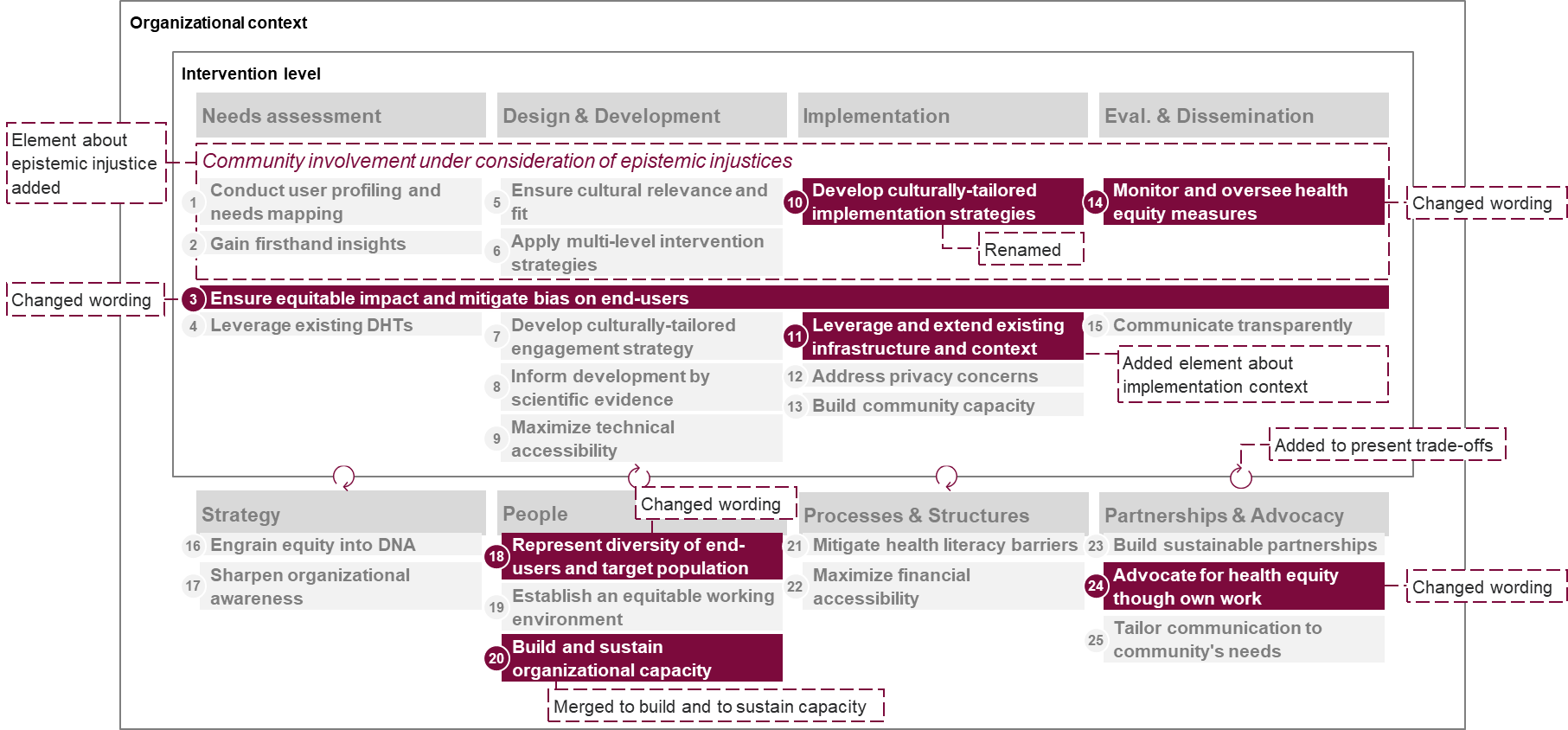


3.3 Final set of the design principles after end-user workshops (Step 3)


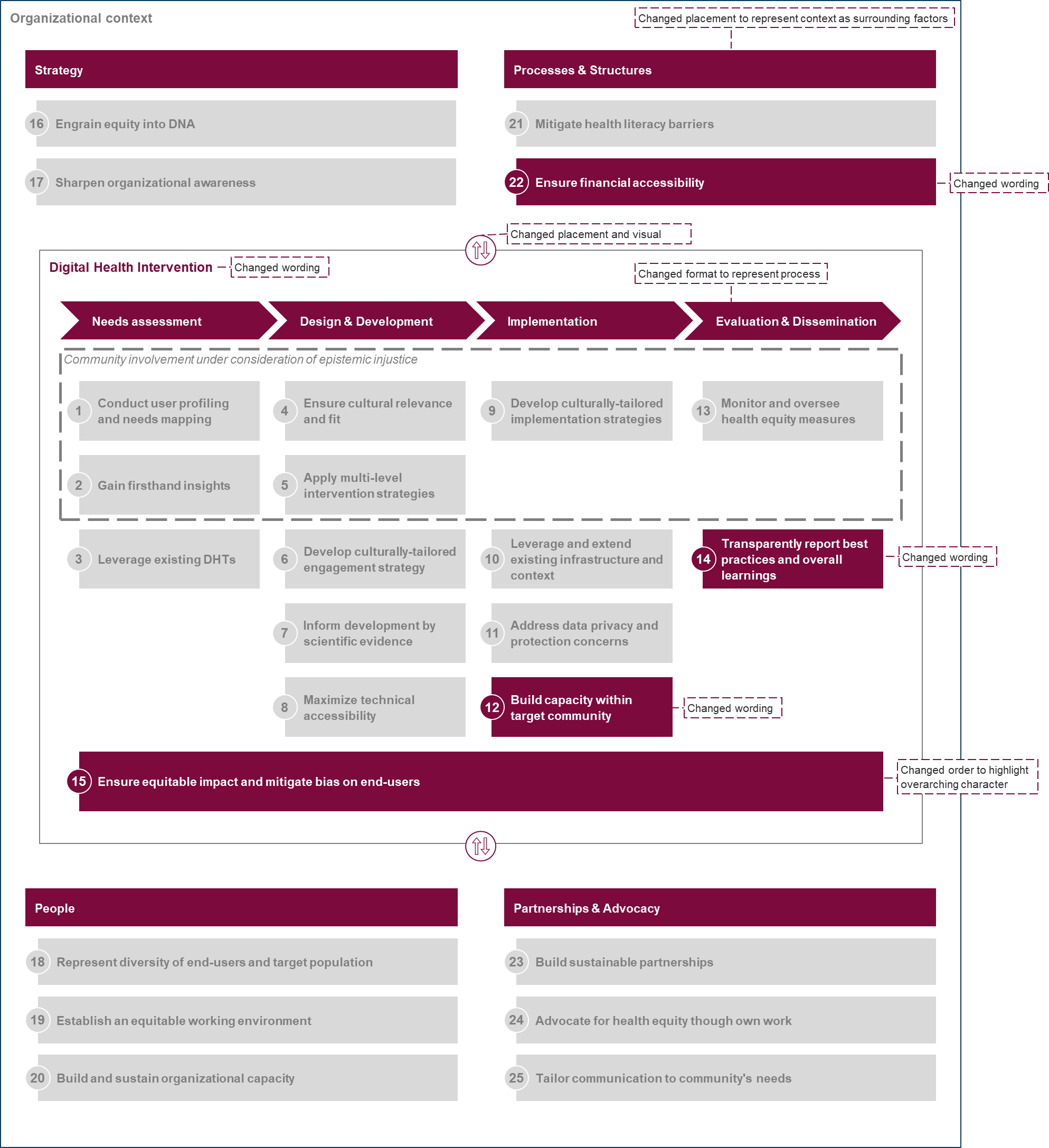


SM.4: Overview of Included Studies/Guidelines

| **Study** | **Title** | **SCOPUS ASJC Overarching category** | **SCOPUS ASJC subcategory** |
| --- | --- | --- | --- |
| Abràmoff et al. (2023) | Considerations for addressing bias in artificial intelligence for health equity | Physical Sciences, Health Sciences | Computer Science, Medicine |
| Adsul et al. (2022) | Grounding implementation science in health equity for cancer prevention and control | Health Sciences | Medicine |
| Alcaraz et al. (2017) | The ConNECT Framework: A model for advancing behavioral medicine science and practice to foster health equity | Social Sciences & Humanities | Psychology |
| Alves-Bradford et al. (2020) | Mental health equity in the twenty-first century: Setting the stage | Health Sciences | Medicine |
| Arrington (2022) | The 5D Cycle for Health Equity: Combining Black Feminism, Radical Imagination, and Appreciative Inquiry to Transform Perinatal Quality Improvement | Health Sciences | Medicine, Nursing |
| Arundell et al. (2020) | Advancing mental health equality: a mapping review of interventions, economic evaluations and barriers and facilitators | Health Sciences | Medicine |
| Aschbrenner et al. (2023) | An Implementation Scientist’s Toolkit for Getting Started with Health Equity-Focused Implementation Research | Physical Sciences | Computer Science |
| Bakken et al. (2019) | Behavioral Interventions Using Consumer Information Technology as Tools to Advance Health Equity | Health Sciences | Sciences |
| Baumann and Cabassa (2020) | Reframing implementation science to address inequities in healthcare delivery | Health Sciences | Medicine |
| Brownson et al. (2021) | Implementation science should give higher priority to health equity | Health Sciences | Medicine |
| Bucknor et al. (2023) | A Framework for Developing Health Equity Initiatives in Radiology | Health Sciences | Medicine |
| Budhwani et al. (2022) | Challenges and strategies for promoting health equity in virtual care: Findings and policy directions from a scoping review of reviews | Health Sciences | Medicine |
| Calancie et al. (2022) | Practical Approaches for Promoting Health Equity in Communities | Health Sciences | Medicine |
| Center for Disease Control and Prevention | Center for Disease Control and Prevention (CDC) CORE commitment to Health Equity | Health Sciences* | *n/a* |
| Dankwa-Mullan et al. (2021) | A Proposed Framework on Integrating Health Equity and Racial Justice into the Artificial Intelligence Development Lifecycle | Health Sciences | Medicine |
| Eslava-Schmalbach et al. (2019) | Conceptual framework of equity-focused implementation research for health programs (EquIR) | Health Sciences | Medicine |
| Fricker (2007) | Epistemic Injustice: Power and the Ethics of Knowing | Social Sciences & Humanities* | *n/a* |
| Glasgow et al. (1999) | Evaluating the Public Health Impact of Health Promotion Interventions: The RE-AIM Framework | Health Sciences | Medicine, Health Professions |
| Grogan (2022) | First Nations Health Equity Strategy - making tracks together: Queensland's Aboriginal and Torres Strait Islander health equity framework | Health Sciences | Medicine |
| Hogan et al. (2018) | Dimensionality and R4P: A Health Equity Framework for Research Planning and Evaluation in African American Populations | Health Sciences | Medicine |
| Holeman & Kane (2019) | Human-centered design for global health equity | Life Science, Social Sciences & Humanities | Neuroscience, Psychology |
| Jaworski et al. (2023) | Advancing digital health equity: Directions for behavioral and social science research | Life Science, Social Sciences & Humanities | Neuroscience, Psychology |
| Kerkhoff et al. (2022) | Addressing health disparities through implementation science-a need to integrate an equity lens from the outset | Health Sciences | Medicine |
| Kolla et al. (2022) | A health equity framework to address racial and ethnic disparities in melanoma | Health Sciences | Medicine |
| Krishnaswami et al. (2019) | Community-Engaged Lifestyle Medicine as a Framework for Health Equity: Principles for Lifestyle Medicine in Low-Resource Settings | Health Sciences | Medicine |
| Loper et al. (2021) | Equity is fundamental to implementation science | Physical Sciences, Health Sciences | Computer Science, Medicine |
| Lopez et al. (2023) | Achieving Health Equity in the Care of Patients with Heart Failure | Health Sciences | Medicine |
| Lyles et al. (2022) | Centering Health Equity in Telemedicine | Health Sciences | Medicine |
| Lyles et al. (2023) | Multilevel Determinants of Digital Health Equity: A Literature Synthesis to Advance the Field | Health Sciences | Medicine |
| Marrone et al. (2022) | Community-Based Participatory Research and Human-Centered Design Principles to Advance Hearing Health Equity | Health Sciences | Medicine, Health Professions |
| Marshall et al. (2012) | A Roadmap and Best Practices for Organizations to Reduce Racial and Ethnic Disparities in Health Care | Health Sciences | Medicine |
| Miller et al. (2023) | Equity and behavioral digital health interventions: Strategies to improve benefit and reach | Life Science, Social Sciences & Humanities | Neuroscience, Psychology |
| Nápoles and Stewart (2018) | Transcreation: an implementation science framework for community-engaged behavioral interventions to reduce health disparities | Health Sciences | Medicine |
| Richardson et al. (2022) | A framework for digital health equity | Physical Sciences, Health Sciences | Computer Science, Medicine |
| Rodriguez et al. (2023) | Thinking Beyond the Device: An Overview of Human- and Equity-Centered Approaches for Health Technology Design | Physical Science, Health Sciences | Medicine, Engineering |
| Seaton et al. (2022) | Community stakeholder‐driven technology solutions towards rural health equity: A concept mapping study in Western Canada | Health Sciences | Medicine |
| Serino-Cipoletta et al. (2022) | Telemedicine and Health Equity During COVID-19 in Pediatric Gastroenterology | Health Sciences | Medicine |
| Shaw et al. (2021) | Recommendations for health equity and virtual care arising from the COVID-19 pandemic: Narrative review | Health Sciences | Medicine |
| Shelton and Brownson (2024) | Enhancing Impact: A Call to Action for Equitable Implementation Science | Health Sciences | Medicine |
| Stiles-Shields et al. (2022) | A Call to Action: Using and Extending Human-Centered Design Methodologies to Improve Mental and Behavioral Health Equity | Life Science, Health Sciences | Medicine, Computer Science, Engineering |
| Trinh-Shevrin, Nadkarni, et al. (2015) | Defining an integrative approach for health promotion and disease prevention: A population health equity framework | Health Sciences | Medicine |
| van Roode et al. (2020) | Values are not enough: qualitative study identifying critical elements for prioritization of health equity in health systems | Health Sciences | Medicine |

SM.5: Exemplary Application of the Design Principles to German Digital Therapeutics Organizations (DiGA)

5.1 Exemplary application of design principles

|  | **Design Principle** | | **DiGA1: Mental health** *Scenario: develop a new anxiety therapy module* | **DiGA2: Acute and chronic back pain** *Scenario: develop a new therapy module focused on managing knee osteoarthritis* |
| --- | --- | --- | --- | --- |
| Design Principle targeting the Digital Health Intervention | 1 | Conduct user profiling and needs mapping | User researchers conduct surveys leveraging existing users, social media announcements and notice boards at physician offices to understand usage patterns across patient demographics and tailor modules to their needs. | User research is conducted leveraging an external provider to engage diverse user groups, e.g., older adults or individuals with limited digital experience, to ensure the module is user-friendly and accessible. |
|  | 2 | Gain firsthand insights | Researchers and product owner collaborate with “Deutsche Depressionshilfe” to gain deeper understanding of lived experiences and gather cultural and contextual feedback for its anxiety module. | Researchers and product owners collaborate with relevant community stakeholders, e.g., Deutsche Schmerzgesellschaft e.V., to gain deeper understanding of lived experiences with chronic pain, common disparities and barriers to access. |
|  | 3 | Ensure equitable impact and mitigate bias on end-users | Leadership appoints trained equity champions and bias officers to challenge potential biases in assumptions, algorithms, and content and perform regular audits | Health equity considerations are baked into every decision-making process as organization-wide standards that are needed to clear the next stage |
|  | 4 | Leverage existing DHIs | The development team analyzes well-regarded apps for anxiety management like Headspace and Calm to understand their strengths and limitations, particularly their features, accessibility, and effectiveness for underrepresented groups. | The team analyzes telehealth services used by orthopedic clinics and reviews well-regarded health and activity apps (e.g., Strava) to understand their strengths and limitations, particularly their features, accessibility, and effectiveness for underrepresented groups. |
|  | 5 | Ensure cultural relevance and fit | The development team sets up an advisory board of therapists, onboarded patients, and statutory health insurance partners to share their experiences with anxiety and discuss the types of support they find helpful and to provide iterative feedback during development | The team sets up a panel consisting of physiotherapists and patients to engage in co-design processes and to provide iterative feedback during development |
|  | 6 | Apply multi-level intervention strategies | They develop a companion app for family members, providing tips on how to support loved ones dealing with anxiety, such as recognizing triggers and reinforcing coping strategies. | They develop a physician facing interface to provide progress reports to the attending physiotherapists to incorporate into in-person rehab program |
|  | 7 | Develop culturally-tailored engagement strategy | The development team leverages user feedback to tailor strategies and develop exercises that are both generalizable and customizable, e.g. including audio-guided exercises, infographics, and interactive journaling to cater to different learning styles, or a progress tracker and feedback system to adjust content based on user behavior. | Instructions are being offered both visually and audio-guided and patients can provide feedback after each exercise on level of intensity, pain sensations etc. to adapt the next training session according to own capabilities. |
|  | 8 | Inform development by scientific evidence | The development team develops therapy modules based on cognitive behavioral therapy (CBT) | Interventions build upon established physiotherapy guidelines (e.g., from Deutsche Verband für Physiotherapie (ZVK) e.V.") and rehabilitation protocols. |
|  | 9 | Maximize technical accessibility | They commit to compatibility for operating systems from the last 8 years for Android, iOS and other major operating systems. | Technical compatibility is ensured for Android systems of the past 9 years and iOS systems of past 5 years. They ensure that the module’s videos and guided exercises can be downloaded for offline use and provide a low-data mode with reduced image quality and no autoplay features to cater to users with limited internet connectivity. |
|  | 10 | Develop culturally-tailored implementation strategies | The sales team collaborates with local clinics and mental health advocates to embed the module into existing community health initiatives. They train healthcare providers to recommend and guide patients through the module, ensuring it becomes part of routine care. | The sales team collaborates with local orthopedic clinics and senior centers to introduce the module to older patients who may not seek digital solutions independently |
|  | 11 | Leverage and extend existing infrastructure and context | The development team offers a standalone, web-based version of the module that clinicians can access via a secure login, avoiding the need for costly EHR integration. The module provides printable summaries for patients and clinicians to discuss in sessions. | The development team enables data-sharing with orthopedic clinics to track patient progress directly in their system to build and adapt the in-person treatment accordingly |
|  | 12 | Address data privacy and protection concerns | The development team integrates GDPR-compliant encryption and updates privacy policies in line with Germany’s digital health regulations. They incorporate user settings to allow individuals to review, download, or delete their data easily to give control to users over their data. | The development team integrates GDPR-compliant encryption and updates privacy policies in line with Germany’s digital health regulations. They incorporate user settings to allow individuals to review, download, or delete their data easily to give control to users over their data. |
|  | 13 | Build capacity within target community | The sales team conducts webinars for healthcare providers, advocacy groups and statutory health insurers to help them explain its modules to eligible patients and leverages collaborations with national mental health organizations (e.g., Deutsche Depressionshilfe) as ambassadors. | The sales team collaborates with advocacy organizations (e.g., Deutsche Schmerzgesellschaft e.V.) to hold seminars with potential patients on the benefits of the therapy module and provides relevant resources and support to increase adoption |
|  | 14 | Monitor and oversee health equity measures | They monitor outcomes across statutory and private insurance and stratified by demographics to identify potential inequities in the module's performance to feed back into its product development decisions. | User researchers monitor improvements in mobility and pain reduction stratified by demographics to identify potential inequities in the module's performance to feed back into its product development decisions. |
|  | 15 | Transparently report best practices and overall learnings | They release publicly accessible transparency reports detailing anonymized usage and outcomes by region and demographics. | Product owners synthesize and publicly report (e.g., through company website, roundtables, conferences) equity-related learnings and best practices to share with peers |
| Decide Principles targeting the Organizational Context | 16 | Engrain equity into DNA | Health equity becomes a key pillar of their business strategy, influencing product roadmaps, team KPIs, and reporting. | They establish a cross-functional "Equity Task Force" to review new products and initiatives through an equity lens. |
|  | 17 | Sharpen organizational awareness | The leadership team conducts diversity audits and establishes a new Diversity, Equity, and Inclusion (DEI) leadership role. | Leadership conducts an internal assessment on how and to what extend equity considerations are currently being factored into product development. |
|  | 18 | Represent diversity of end-users and target population | Human resources partners with diversity-focused organizations, community groups, and job boards to reach underrepresented communities during hiring process | Human Resources has dedicated DEI targets to ensure diverse representation of talents. |
|  | 19 | Establish an equitable working environment | Leadership sets up anonymous reporting and feedback channels for incidents of bias, discrimination or overall feedback for improvement on health equity action and commit to investigating each report transparently | Leadership creates and establishes a clear policy framework for inclusive workplace practices and accountability measures. |
|  | 20 | Build and sustain organizational capacity | Leadership regularly invites guest speakers, such as community health workers or patient advocates, to share lived experiences and actionable insights for bi-directional learning. | Leadership requires all employees to undergo yearly training sessions on topics like unconscious bias and accessibility in digital health. |
|  | 21 | Mitigate health literacy barriers | The customer support implements live chat or (video-)phone support to assist users in navigating the platform. | The sales team and customer support provide multilingual video walkthroughs and FAQs on its website and app for less tech-savvy users |
|  | 22 | Ensure financial accessibility | Leadership works with insurers to ensure reimbursement is simple and widely available for all covered patients. | Leadership works with insurers to ensure reimbursement is simple and widely available for all covered patients. |
|  | 23 | Build sustainable partnerships | Leadership establishes long-term partners (e.g., Deutsche Depressionshilfe) to foster bi-directional learning and amplify mental health equity | Leadership establishes Deutsche Schmerzgesellschaft e.V. as long-term partner to foster bi-directional learning and amplify health equity in the back pain therapy. |
|  | 24 | Advocate for health equity though own work | All employees take up opportunities to join mental health and health equity roundtables, speaks on conferences and collaborates on awareness campaigns. | All employees take up opportunities to join chronic pain and health equity roundtables, speaks on conferences and collaborates on awareness campaigns. |
|  | 25 | Tailor communication to community's needs | They create a dedicated "Equity & Inclusion" section on their website featuring blogs, videos, and infographics tailored to diverse population, where they publish content addressing systemic mental health disparities and offer culturally relevant mental health tips. | They create a dedicated "Equity & Inclusion" section on their website featuring blogs, videos, and infographics tailored to diverse population, where they publish content addressing systemic health disparities and offer culturally relevant tips on therapeutic training. |

5.2 Current level of implementation of design principles and major roadblocks

*Note: o – not implemented; + – partially implemented; ++ – fully implemented*

|  |  |  | **Current level of implementation** | |  |
| --- | --- | --- | --- | --- | --- |
|  |  | **Principle Name** | ***DiGA1****: Mental health* | ***DiGA2:*** *Acute and chronic back pain* | **Key Roadblocks** |
| Design Principles targeting the Digital Health Intervention | 1 | Conduct user profiling and needs mapping | + | + | Available resources (time, money), access to diverse user groups, data protection laws |
|  | 2 | Gain firsthand insights | + | o | Access to user groups, capacity to build sustainable partnerships, time-to-market expectations |
|  | 3 | Ensure equitable impact and mitigate bias on end-users | + | + | Makes product more expensive, longer time-to-market, missing equity capabilities |
|  | 4 | Leverage existing DHIs | ++ | + | DiGAs only accessible via code, regulation harmonizes many features |
|  | 5 | Ensure cultural relevance and fit | + | + | Access to diverse user groups |
|  | 6 | Apply multi-level intervention strategies | o | o | Available budget |
|  | 7 | Develop culturally-tailored engagement strategy | + | ++ | Customization options limited within DiGA regulations |
|  | 8 | Inform development by scientific evidence | ++ | ++ | Updating DiGAs based on new scientific evidence triggers new accreditation process |
|  | 9 | Maximize technical accessibility | + | + | Offline or low-data options not feasible or not priority until now |
|  | 10 | Develop culturally-tailored implementation strategies | o | + | available resources (highly labor intense) |
|  | 11 | Leverage and extend existing infrastructure and context | o | o | Heterogeneity of technical infrastructure |
|  | 12 | Address data privacy and protection concerns | ++ | ++ | n/a |
|  | 13 | Build capacity within target community | o | o | available resources (time, personnel, money) |
|  | 14 | Monitor and oversee health equity measures | o | o | Limited capacity and capabilities, German data protection and privacy laws limits collection of stratified data, |
|  | 15 | Transparently report best practices and overall learnings | o | o | Prior low awareness for and prioritization of health equity action, available resources (time, personnel) |
| Design Principles targeting the Organizational Context | 16 | Engrain equity into DNA | o | o | Competing interests due to resource constraints, limited awareness and prioritization of dedicated health equity action |
|  | 17 | Sharpen organizational awareness | o | o | Competing interests due to resource constraints, limited awareness and prioritization of dedicated health equity action |
|  | 18 | Represent diversity of end-users and target population | + | + | Low priority due to inherently diverse team (esp. in engineering roles) |
|  | 19 | Establish an equitable working environment | + | + | Lack of institutionalization due to competing interests |
|  | 20 | Build and sustain organizational capacity | o | o | Lack of resources (time, money, personnel) and competing interests (e.g., fast time-to-market) |
|  | 21 | Mitigate health literacy barriers | + | + |  |
|  | 22 | Ensure financial accessibility | ++ | ++ | n/a |
|  | 23 | Build sustainable partnerships | + | o | Capacity to build sustainable partnerships |
|  | 24 | Advocate for health equity though own work | o | o | Competing interests due to resource constraints, limited awareness and prioritization of dedicated health equity action |
|  | 25 | Tailor communication to community's needs | o | o | Competing interests due to resource contraints, limited awareness and prioritization of dedicated health equity action |

Looking at the current level of implementation across both DiGAs, only four out of the 25 design principles are fully implemented as of today, with the rest being partially implemented (~40%) or not at all implemented (44% – 48%). This becomes even more prevalent when looking at design principles for the organizational context, where only one out of 10 principles was fully implemented (*“maximize financial accessibility”*) which was pre-determined by the regulatory context of this application. Key roadblocks mentioned across both organizations for intervention level principles were resource constraints, i.e., time, budget, personnel, capabilities, access to diverse user groups, regulatory constraints, and competing interests of fast time-to-market. For organization level principles key roadblocks across both organizations were limited awareness and prioritization of dedicated health equity action, especially given competing interests for making a successful business case.

SM.6: Design Principle Details

|  | **Name** | **Aim** | **Imple-menter** | **Context** | **Mechanism** | **Sub-Mechanism** | **Rationale** | **Supporting sources** |
| --- | --- | --- | --- | --- | --- | --- | --- | --- |
| 1 | **Conduct user profiling and needs mapping** | To inform the **development of tailored strategies and resources for inclusive intervention design and implementation** | Researchers designers, and developers | Needs assessment | by conducting comprehensive assessments that identify diverse user groups and their specific needs, including barriers related to access, digital literacy, and disparities. | **1. Data collection** Conceptually outline the assessment approach to identify characteristics and needs of diverse user groups as well as barriers to access, literacy, and disparities.  Ideally, involve target communities in defining data measures to ensure meaningful data collection.  To accurately reflect health disparities and needs by collecting data stratified by race, ethnicity, and social determinants. Gather the required data to perform the outlined assessment. Prioritize ethical data practices and data quality. Implement robust data governance mechanisms and ensure diversity in sampling.  **2. Data Analysis:** Analyze assessment data to identify common themes and specific needs of diverse user groups.   **3. Tailored Strategies:** Translate findings to develop intervention strategies and resources that address the identified needs and barriers. Ensure these strategies are inclusive and consider the diverse characteristics of user groups. | By conducting comprehensive assessments, this principle ensures that the needs of diverse user groups are identified and addressed. This approach helps in developing tailored strategies and resources that enhance the inclusivity and effectiveness of the intervention. | Bakken et al. (2019), Miller et al. (2023), Nápoles and Stewart (2018), Loper et al. (2021), Abràmoff et al. (2023), Lopez et al. (2023), Jaworski et al. (2023), Lyles et al. (2022), Krishnaswami et al. (2019), Hogan et al. (2018), Grogan (2022), Calancie et al. (2022), Alcaraz et al. (2017), Marshall et al. (2012), Eslava-Schmalbach et al. (2019), Arrington (2022), Williams et al. (2023), Dankwa-Mullan et al. (2021) |
| 2 | **Gain firsthand insights** | To **gain firsthand, in-depth insights into disparities, root causes, and contextual realities** | Researchers designers, and developers | Needs assessment | by actively involving community stakeholders using participatory research and co-design methodologies. | **1 Stakeholder Involvement:** Identify key community stakeholders to involve ensuring representation of the community. Actively engage community stakeholders in the research and development process through trust and relationship building as well as continuous bilateral support. Organize focus groups, interviews, and workshops with community members.  **2 Gather and synthesize insights:** Collect detailed information about disparities, root causes, and contextual realities. Synthesize findings to inform development strategies. | By involving community stakeholders, this principle ensures that interventions are informed by firsthand insights into disparities and contextual realities. This approach enhances the relevance and effectiveness of the intervention. | Nápoles and Stewart (2018), Loper et al. (2021), Abràmoff et al. (2023), Lyles et al. (2022), Krishnaswami et al. (2019), Hogan et al. (2018), Grogan (2022), Calancie et al. (2022), Dankwa-Mullan et al. (2021) |
| 3 | **Leverage existing DHIs** | To **build upon existing, relevant digital health technologies** | Developers researchers community leaders | Needs assessment | by assessing and adapting interventions systematically and transparently to meet the needs of diverse populations, guided by established frameworks. | **1 Assess Adaptability:** Conduct a thorough market research to identify existing evidence-based interventions for similar health contexts but different target populations.  Conduct a comprehensive overview of established adaptation frameworks and successful past cultural adaptations, synthesizing key success factors. Based on the insights, evaluate the adaptability of existing and/or new interventions for target populations.  **2 Systematic Adaptation:** Conduct adaptations systematically using established frameworks. Utilize a common data platform to track adaptations across contexts and populations. Ensure transparency in the adaptation process and communicate all changes and rationales to all relevant stakeholders. | By systematically and transparently adapting interventions, this principle ensures that interventions remain relevant and effective for diverse populations. This approach enhances trust and accountability in the adaptation process. | Bakken et al. (2019), Miller et al. (2023), Nápoles and Stewart (2018), Baumann and Cabassa (2020), Dankwa-Mullan et al. (2021) |
| 4 | **Ensure cultural relevance and fit** | To **ensure relevance, fit, and practicality and mitigate biases** | Researchers designers, developers, community members | Design and development | by utilizing participatory approaches throughout the entire intervention development process. | **1 Engage Stakeholders:** Identify a comprehensive, representable list of stakeholders  Involve community members, researchers, and developers from the outset. Facilitate participatory workshops and design sessions.  **2 Co-Design Process:** Collaboratively develop intervention components, ensuring they are practical and relevant. Use iterative feedback to refine and improve the intervention.  **3 Bias Mitigation:** Identify and address potential biases in the development process. Ensure diverse representation among stakeholders. | By utilizing participatory approaches, this principle ensures that interventions are relevant, practical, and free from biases. This approach enhances the overall quality and effectiveness of the intervention. | Grogan (2022), Baumann and Cabassa (2020), Loper et al. (2021), Arundell et al. (2020) |
| 5 | **Apply multi-level intervention strategies** | To develop intervention strategies that **target various levels of influence** from the patient and clinician to the microsystem (families, clinical teams), organization, community, and policy levels. | Researchers developers policymakers healthcare providers. | Design and development | by leveraging scientific evidence to inform multi-level intervention strategy | **1 Multi-Level Targeting:** Conduct a comprehensive review of scientific literature and existing evidence on multi-level intervention strategies and assess applicability for own intervention design.  If applicable, leverage data and scientific evidence to inform own multi-level intervention strategy.   **2 Integration and Alignment:** Ensure that strategies are integrated across different levels and aligned with overarching health equity goals. Collaborate with stakeholders at each level to refine and implement strategies. | By using scientific, innovative, and data-driven strategies that target multiple levels of influence, this principle ensures that interventions are comprehensive and effective. This approach addresses the complex and interconnected factors influencing health equity. | Trinh-Shevrin, Nadkarni, et al. (2015), Lyles et al. (2023), Baumann and Cabassa (2020), Loper et al. (2021), Abràmoff et al. (2023), Dankwa-Mullan et al. (2021) |
| 6 | **Develop culturally-tailored engagement strategy** | To **fit population preferences and to sustain user engagement** | Designers / developers | Design and development | by tailoring content and behavior change mechanisms to user characteristics such as culture, language, learning styles, functional, digital, and health literacy. | **1 User Profiling:** Identify and document user characteristics such as culture, language, learning styles, and literacy levels. If necessary, create user personas representing different segments of the user population  **2 Tailor Content:** Develop content and behavior change mechanisms tailored to these characteristics. In case of highly diverse user groups, consider to allow for adaptive content and change behavior mechanisms.  Ensure that content is accessible and understandable for users with varying literacy levels.  **3 Engagement Strategies:** Create engagement frameworks outlining strategies to sustain user engagement. Consider factors such as user preferences, motivational triggers, barriers to engagement and mitigation strategies, e.g., multi-channel engagement Implement strategies to sustain user engagement based on their preferences. Consider factors such as integrated care options, involvement of family members, community members and/or care providers.  Use feedback loops to continuously improve and tailor content. | By considering user characteristics, this principle ensures that the intervention content is relevant and engaging for the target population. This approach helps in sustaining user engagement and improving the effectiveness of the intervention. | Center for Disease Control and Prevention, Lopez et al. (2023) |
| 7 | **Inform development by scientific evidence** | To **develop intervention components, delivery methods, and strategies** that are **informed by scientific evidence of effectiveness in disparity populations.** | Researchers designers, and developers | Design and development | by building upon findings from evidence-based interventions and locally relevant programs | **1 Review Scientific Evidence:** Conduct a comprehensive review of scientific literature and existing evidence-based interventions, focusing on effective strategies in similar health contexts and for disparity populations Gather data from locally relevant programs, including qualitative and quantitative outcomes.  **2 Synthesis of findings:** Identify key components and strategies that have been proven effective. Analyze the mechanisms of action that make these components effective in different contexts.  **3 Develop Components:** Design intervention components based on the identified evidence. Ensure that delivery methods and strategies align with the needs and contexts of disparity populations.  **4 Test and Refine:** Pilot the intervention components and gather feedback from target users. Refine the components based on feedback and ongoing evidence review. | By developing interventions informed by scientific evidence, this principle ensures that the strategies and methods used are effective for disparity populations. This approach enhances the likelihood of success and improves health outcomes for these groups. | Dankwa-Mullan et al. (2021), Arundell et al. (2020), Bakken et al. (2019), Lyles et al. (2023), Marshall et al. (2012), Miller et al. (2023), Nápoles and Stewart (2018), Baumann and Cabassa (2020), Budhwani et al. (2022), Lopez et al. (2023), Lyles et al. (2022), Krishnaswami et al. (2019), Hogan et al. (2018), Grogan (2022), Alves-Bradford et al. (2020), Calancie et al. (2022), Shaw et al. (2021), Serino-Cipoletta et al. (2022), Arrington (2022), Alcaraz et al. (2017), Richardson et al. (2022) |
| 8 | **Maximize technical accessibility** | To maximize technical accessibility of developed intervention | Designers / developers | Design and development | by ensuring digital interventions are agnostic to devices and operating systems, mindful of wifi and cellular data availability, and usable by multiple people under one account. | **1 Cross-Platform Compatibility:** Design interventions to be compatible with various devices (e.g., smartphones, tablets, computers) and operating systems (e.g., iOS, Android, Windows), including older / outdated operating systems.  **2 Data Accessibility:** Optimize interventions for use with limited wifi, cellular data and overall limited network coverage (e.g., in rural areas). Provide offline functionality where possible   **3 Multi-User Accounts:** Allow multiple users to access the intervention through a single account and factor it into engagement strategies and their implications.  Ensure data privacy and security while facilitating shared use. | By designing digital interventions to be device and data agnostic, this principle ensures that they are accessible to a broader audience, regardless of their technological limitations. This approach promotes inclusivity and maximizes the reach of the intervention. | Nápoles and Stewart (2018), Trinh-Shevrin, Nadkarni, et al. (2015) |
| 9 | **Develop culturally-tailored implementation strategies** | **To ensure widespread adoption of interventions** | developers community leaders policymakers | Implementation | by developing tailored implementation strategies for vulnerable communities, incorporating cultural competence, advocacy, and focus on increasing access to services. | **1 Acknowledge Limitations:** Involve key stakeholders from the outset and facilitate collaborative assessment, development, and planning sessions. Conduct a comprehensive overview of existing implementation strategies including their context, target population, and success indicators.  Identify and acknowledge the limitations of existing implementation strategies when targeting marginalized populations. Focus explicitly on contextual factors and structural barriers that might hinder adoption.   **2 Tailored Strategies:** Co-develop implementation strategies, policies, and resources tailored to the needs and priorities of the target communities.  **3 Sustainability Planning:** Create plans to sustain interventions within the community. Monitor and adjust based on stakeholder feedback and evolving needs. | By tailoring implementation strategies to the needs of vulnerable communities, this principle ensures that interventions are more likely to be adopted and effective. This approach addresses specific barriers and enhances the inclusivity and reach of the interventions. | Dankwa-Mullan et al. (2021), Miller et al. (2023), Budhwani et al. (2022), Richardson et al. (2022) |
| 10 | **Leverage and extend existing infrastructure and context** | **To facilitate the delivery, sustainability, and equitable dissemination of interventions** | Healthcare administrators IT specialists designers | Implementation | by strategically integrating technological tools and infrastructure within healthcare settings. | **1 Identify Technological Needs and overarching context:** Assess the status quo of relevant infrastructure and derive technological needs within healthcare settings to support intervention delivery. Consider the wider implementation context, e.g., how (in-)voluntary is the DTI usage or how do more or less sophisticated systems perform in different social settings, and derive different strategies to support intervention delivery and adoption  **2 Integrate Tools and Infrastructure:** Leverage existing technological tools such as mHealth applications and social media platforms to facilitate intervention dissemination. Ensure necessary resources and technology are accessible to end user as well as supporting infrastructure stakeholders.   **3 Multilevel Approaches:** Utilize multilevel strategies to integrate technology at different levels (individual, organizational, community). Provide training and support for effective use where needed. | By strategically integrating technology, this principle ensures interventions are sustainable and can be equitably disseminated across diverse healthcare settings. This approach enhances the overall impact and accessibility of the interventions. | Marshall et al. (2012), Baumann and Cabassa (2020), Loper et al. (2021), Budhwani et al. (2022), Jaworski et al. (2023) |
| 11 | **Address data privacy and protection concerns** | To **address user privacy concerns comprehensively** | Researchers policymakers healthcare administrators | Implementation | by ensuring protection at the individual study level, continuous monitoring, and through policy advocacy at institutional, state, and national levels. | **1 Individual Study Level:** Implement robust privacy protection measures in individual studies following or exceeding relevant standards and guidelines. Ensure informed consent processes clearly explain privacy protocols Prioritize transparency of privacy protection methods to gain trust. Anticipate user privacy concerns of target audience, taking historic disparities and context into account, and address proactively and transparently  **2 Continuous Monitoring:** Regularly review and update privacy measures to address emerging concerns. Engage with participants to gather feedback on privacy concerns or potential issues.   **3 Policy Advocacy:** Leverage feedback processes and collaboration opportunities to exchange best practices with peers and other stakeholders and to advocate for relevant privacy policies at institutional, state, and national level. | By addressing privacy concerns at multiple levels, this principle ensures that interventions protect user data and build trust. This approach supports ethical research practices and enhances participant engagement. | Miller et al. (2023), Nápoles and Stewart (2018), Budhwani et al. (2022), Bakken et al. (2019) |
| 12 | **Build capacity within target community** | To **build community capacity for intervention dissemination** | Community leaders healthcare administrators developers | Implementation | by hiring and training community members, providing necessary resources and support, and establishing feedback systems. | **1 Hire and Train:** Identify key community stakeholders that would make strategic partners during implementation and dissemination.  Hire key community members to support intervention dissemination. Think about the right incentive structure, provide comprehensive training and fair compensation.  **2 Resources and Support:** Offer continuous learning materials, manuals, and technical support. Co-develop collaboration models and support options to continuously increase community capacity and capabilities.   **3 Feedback Systems:** Establish systems to gather and incorporate community feedback continuously.  Leverage feedback to evolve and improve interventions as well as approach for community capacity | By building community capacity, this principle ensures that interventions are disseminated effectively and sustainably. This approach leverages local knowledge and resources, enhancing the intervention's acceptance and impact. | Budhwani et al. (2022), Bakken et al. (2019) |
| 13 | **Monitor and oversee health equity measures** | To **ensure continuous health equity improvement of developed intervention** | Researchers evaluators developers | Evaluation and dissemination | select and validate equity-focused outcome measures for the target population and implement structured assessments for monitoring. | **1 Select Outcome Measures:** Choose equity-focused outcome measures based on established conceptual frameworks.  **2 Validate Measures:** Validate selected measures for the target population with key stakeholders and adapt as appropriate to reflect the needs and priorities of the target community.  **3 Structured Assessments:** Implement structured assessments and data collection strategies. Monitor outcomes continuously and adjust as needed.  **4 Feedback loop for continuous improvement** Establish continuous feedback loops to operationalize health equity improvements on the DHI based on outcome measures and assessment | By focusing on equity in outcome measurement, this principle ensures that interventions are evaluated fairly and comprehensively. This approach supports the identification of meaningful and relevant outcomes for diverse populations. | Nápoles and Stewart (2018) |
| 14 | **Transparently report best practices and overall learnings** | **To avoid repetition of unintended consequences** | Researchers evaluators developers | Evaluation and dissemination | by ensuring transparency and overall rigorous adherence to reporting standards. | **1 Adhere to Standards:** Follow established reporting standards, especially with an equity focus (e.g., PRISMA guidelines).  **2 Report Unintended Consequences and Continuous Improvement** Document and report any unintended consequences of interventions that might exacerbate the (digital) health divide or health disparities Use reported data to inform and improve future interventions and to avoid repeating past mistakes. Interact with peers and other relevant stakeholders to facilitate bi-directional learning | By adhering to rigorous reporting standards and being transparent about unintended consequences, this principle ensures accountability and continuous improvement in intervention development. This approach fosters trust and enhances the quality of research. | Nápoles and Stewart (2018), Eslava-Schmalbach et al. (2019), Abràmoff et al. (2023), Dankwa-Mullan et al. (2021) |
| 15 | **Ensure equitable impact and mitigate bias on end-users** | Ensure equitable access and impact on end-users | Researchers evaluators developers | All stages | by rigorously evaluating interventions across all stages of development | **1 Rigorous Evaluation:** Conduct thorough evaluations at each stage of intervention development. Define equity-specific markers and indicators to track from the outset.  **2 Alternative Study Designs:** Explore and implement alternative study designs to ensure robust evaluation. **3 Assess Access , Bias and Impact:** Continuously evaluate equitable access and impact on end-users and use findings to refine and improve interventions. Allocate dedicated resources to continuously identify and address potential for bias throughout each step of DHI design | By rigorously evaluating interventions and considering equitable access and impact, this principle ensures that interventions are fair, effective, and continuously improved. This approach supports comprehensive and reliable evaluation practices. | Miller et al. (2023), Bakken et al. (2019) |
| 16 | **Engrain equity into DNA** | To **make health equity a strategic priority** for the organization | Healthcare administrators, quality improvement teams, and equity officers. | Strategy | by centering equity in organization's strategic goals, processes and structures, teams, and initiatives | **1 Strategic goals** Integrate health equity as a core strategic goal in the organization's mission and vision statements. Derive actionable plans with clear objectives and metrics to achieve health equity goals. Role model prioritizing health equity by allocating sufficient resources (time, money) to health equity initiatives.  **2 Processes, structures, and teams** Incorporate equity into all theoretical approaches, processes, and quality improvement efforts. Leverage established theoretical frameworks and lived experiences where possible.  Integrate equity into organizational structures, i.e., align roles, responsibilities, and governance structures  Form teams with diverse backgrounds and expertise. Utilize team members' varied theoretical approaches and lived experiences. Foster a culture of open discussion, experience sharing, and bi-directional learning.   **3 Continuous Monitoring and Reporting** Implement systems to continuously monitor progress towards health equity goals. Regularly evaluate initiatives against predefined equity metrics and implement systematic interventions to reduce disparities. Establish accountability mechanisms to ensure organizational commitment and progress. Transparently report progress and challenges to stakeholders, including staff, patients, and the community. | Embedding health equity principles into quality improvement ensures that all efforts contribute to reducing disparities and promoting fairness in healthcare delivery. | Lopez et al. (2023), Lyles et al. (2022), Calancie et al. (2022), Shaw et al. (2021), Williams et al. (2023), Marshall et al. (2012), Eslava-Schmalbach et al. (2019), Center for Disease Control and Prevention, Jaworski et al. (2023), Arundell et al. (2020), Seaton et al. (2022), Grogan (2022), Bucknor et al. (2023) |
| 17 | **Sharpen organizational awareness** | To **enhance organizational awareness and impact on health equity** | Executive leadership, human resources, and diversity officers. | Strategy  Applicable during strategic planning, organizational assessments, and policy development. | by understanding workforce composition and decision-making processes. | **1 Understand Workforce and Partnership Composition:** Analyze and understand the diversity of the workforce. Identify and evaluate organizational partners and contracted entities. Synthesize findings to get comprehensive understanding of how current composition supports ambition to advance health equity, including potential blind spots and biases  **2 Decision-Making Insights:** Gain insight into decision-making processes, governance structures and their impact on health equity advancement | Understanding workforce composition and decision-making processes helps organizations identify areas for improvement, align policies with equity goals, and create a more inclusive environment. | Calancie et al. (2022) |
| 18 | **Represent diversity of end-users and target population** | To **attract and engage talent from marginalized populations** to represent the diversity of its customers | Human resources and recruitment teams. | People | through improved recruitment processes and strategies. | **1 Revise Recruitment Processes:** Rethink and enhance attraction and recruitment processes. Strategically assess current recruitment processes to identify and eliminate potential bias. Develop strategies specifically aimed at engaging talent from marginalized populations. Implement engagement and outreach initiatives to build relationships with marginalized communities.  **2 Leadership Diversity:** Ensure diversity at all levels, including board and executive levels. Implement training and mentorship programs to develop future diverse leaders. | Inclusive recruitment strategies ensure a more diverse and representative workforce, which can enhance organizational effectiveness and foster a culture of inclusivity. | Lyles et al. (2022), Krishnaswami et al. (2019), Marshall et al. (2012), Grogan (2022) |
| 19 | **Establish an equitable working environment** | To **create an equitable working environment** | Executive leadership, diversity officers, and human resources. | People | by challenging assumptions, adopting a zero-tolerance culture towards racism, addressing power imbalances, and fostering open discussions. | **1 Conduct Assessments:** Perform regular, thorough assessments of organizational practices and policies. Identify power imbalances within the organization and in its interactions with communities. Use data-driven insights to identify systemic exclusion, institutional racism, and health inequities. Regularly monitor and evaluate the effectiveness of anti-racism initiatives and policies, making adjustments as needed.  **2 Policy Development:** Develop and implement a zero-tolerance policy towards racism, clearly outlining consequences for violations. Promote a cultural shift by integrating anti-racism principles into organizational values, practices, and decision-making processes.  **3 Open Discussions:** Facilitate regular open discussions and forums on racism, power imbalances, or other aspects perpetuating disparities, encouraging staff to share experiences and perspectives. Establish support systems for individuals who experience or witness racism, including counseling and reporting mechanisms. | Adopting a zero-tolerance culture towards racism and encouraging open discussions fosters an inclusive and equitable environment, improving the experiences of both staff and patients. | Calancie et al. (2022), Arrington (2022), Williams et al. (2023), Bucknor et al. (2023), Hogan et al. (2018), Shaw et al. (2021), Grogan (2022) |
| 20 | **Build and sustain organizational capacity** | To **build organizational capacity for health equity action** | Training and development teams, diversity officers, and organizational leadership. | People | through regular, mandatory trainings on cultural competency, anti-racism, unconscious bias, and disparities, through bi-directional learning with community health workers and advocates, as well as overall effective allocation and management of institutional resources | **1 Regular Mandatory Trainings:** Conduct regular, mandatory trainings for all staff, including leadership and management boards. Consider cross-training programs to build diverse skill sets. Utilize evidence-based strategies and frameworks for training content. Cover cultural competency, anti-racism, unconscious bias, and intersectional disparities. Consider incentive structure to make these trainings a priority for all staff.  **2 Bi-Directional Learning:** Employ community health workers and cultural/community advocates, either as outside council or full-time employees. Set up dedicated structures and formats to facilitate bi-directional learning between staff and community health workers. **3 Inspire and Leverage Motivation:** Develop a compelling story to communicate the importance of health equity to inspire enthusiasm. Role model health equity action to inspire followship.  Identify and leverage exiting staff motivation to drive health equity actions across the organization, e.g., key strategic allies that can function as multipliers. Develop a convincing incentive structure to provide support and recognition for staff efforts in promoting health equity. **4 Resource Allocation:** Manage and allocate institutional resources effectively to sustain ongoing health equity initiatives. Make health equity initiatives a strategic priority to ensure sufficient (financial) support within the organization. | Mandatory equity trainings enhance staff awareness and skills, fostering a more inclusive and equitable workplace environment. | Arundell et al. (2020), Seaton et al. (2022), Krishnaswami et al. (2019), Grogan (2022), Alves-Bradford et al. (2020), Shaw et al. (2021), Serino-Cipoletta et al. (2022), Alcaraz et al. (2017), Marshall et al. (2012), Budhwani et al. (2022), Bucknor et al. (2023), Eslava-Schmalbach et al. (2019), Center for Disease Control and Prevention, Kolla et al. (2022) |
| 21 | **Mitigate health literacy barriers** | To **increase literal access to care** | Healthcare providers, digital health experts, and community organizations. | Processes and Structures | by developing and providing the necessary health literacy support to engage effectively with healthcare services | **1 External Health literacy programs** Identify available health literacy programs that address different competence levels Identify partnership opportunities to connect customers with these programs   **2 Internal health literacy support** Develop additional support programs based on identified gaps in externally available programs and/or specifically tailored to own digital health offering (e.g., step by step tutorials, interactive user manuals) Ensure (digital) health literacy support is available through multiple channels, e.g., through the health intervention (in-app), a dedicated support website and/or support hotlines / chatbots Create resources to support patient navigation and offer patient navigation services to assist with care access. | Providing comprehensive health literacy support helps patients navigate the healthcare system effectively, improving their health outcomes and reducing disparities in healthcare engagement. | Arundell et al. (2020), Lyles et al. (2022), Seaton et al. (2022), Shaw et al. (2021), Serino-Cipoletta et al. (2022), Marshall et al. (2012), Budhwani et al. (2022), Kolla et al. (2022), Krishnaswami et al. (2019), Jaworski et al. (2023) |
| 22 | **Ensure financial accessibility** | To **make own services financially accessible** | Executive leadership, financial officers, and policy advocates. | Processes and Structures | secure sustainable funding and implement in public or safety net settings | **1 Funding Strategy Development:** Develop a comprehensive healthcare funding strategy that blends private and public investment. Build partnerships with private investors, government agencies, and philanthropic organizations to secure diverse funding sources. Regularly evaluate the effectiveness of funding strategies and report outcomes to stakeholders.  **2 Implementation Support:** Ensure that health initiatives are effectively implemented in public or safety net settings to reach underserved populations. Where applicable, seek accreditation for costs of interventions to be covered by health insurance (e.g., DIGA in Germany)  **3 Policy Advocacy:** Advocate for policies that support universal access to healthcare, emphasizing equity and inclusivity. Engage with policymakers, community leaders, and healthcare providers to build support for universal healthcare policies. Take part in public awareness campaigns to highlight the importance of universal healthcare access and mobilize community support. | Advocating for comprehensive healthcare funding ensures the sustainability and effectiveness of health services, particularly for marginalized populations, by leveraging diverse financial resources and demonstrating their impact. | Lyles et al. (2023), Marshall et al. (2012), Lopez et al. (2023), Lyles et al. (2022), Calancie et al. (2022), Shaw et al. (2021), Arrington (2022), Grogan (2022) |
| 23 | **Build sustainable partnerships** | To **build sustainable community partnerships** | Community engagement teams, organizational leadership, and partnership coordinators. | Partnerships and Advocacy | by actively involving and empowering stakeholders and partnering with diverse community-based organizations based on shared goals, trust, and mutual respect | **1 Stakeholder Involvement:** Actively involve stakeholders at every stage of health initiatives. Partner with community-based organizations across sectors and disciplines.  **2 Shared Goals, Trust, and Respect:** Establish clear shared goals and purposes for partnerships. Focus on mutual benefits and bi-directional learning and support. Commit to long-term engagement and collaboration. Build trust and mutual respect through understanding of priorities, needs, and constraints.  **3 Empower Communities:** Develop community capacity through training, support, and resources. Invest in capacity-building initiatives to strengthen community leadership and participation. Develop programs that foster agency and empower individuals and communities to take action and make decisions. Provide resources and support for communities to pursue their own goals and priorities. | Sustainable community partnerships enhance the effectiveness of health equity initiatives by aligning efforts with community needs and fostering trust and collaboration. | Grogan (2022), Calancie et al. (2022), Williams et al. (2023), Krishnaswami et al. (2019), Lopez et al. (2023), Arundell et al. (2020), Lyles et al. (2022), Shaw et al. (2021), Marshall et al. (2012), Eslava-Schmalbach et al. (2019), Center for Disease Control and Prevention, Bucknor et al. (2023) |
| 24 | **Advocate for health equity though own work** | To **establish strong networks, enhance credibility, and create visibility** for own health equity initiatives | Organizational leadership, policy advocates, and community engagement officers. | Partnerships and Advocacy | by engaging with local boards, community groups, and political representatives. | **1 Policy Advocacy** Identify and map key stakeholders, including local boards, health-oriented community groups, mayors, and congressional representatives. Develop relationships with policymakers to advocate for health equity and influence public policy. Establish and maintain strong networks to support collaborative efforts and resource sharing. Actively serve on and engage with local boards and community groups to influence decision-making and align organizational goals with community needs. Participate in public forums, community meetings, and advocacy events to enhance the organization's visibility and credibility. | Active engagement with community and policy stakeholders fosters trust, enhances the organization's influence, and supports the advancement of health equity goals through collaborative efforts. | Krishnaswami et al. (2019) |
| 25 | **Tailor communication to community's needs** | To **prioritize equity and inclusivity in communication strategies** | Communication teams, community engagement officers, and diversity officers. | Partnerships and Advocacy | by having dedicated resources on equity, inclusion, racism, systematic failures etc. that are tailored to the community's needs. | **1 Community Needs Assessment:** Conduct assessments to understand the communication needs and preferences of the community.  **2 Resource Development:** Create educational materials, support services, and tools to help create visibility for continuous disparities, ongoing efforts etc.  Tailor communication strategies to different community segments, ensuring accessibility and relevance. Ensure that communication materials and strategies are culturally competent and respectful. Use multiple communication channels (e.g., social media, community events, printed materials) to reach diverse audiences. Establish feedback mechanisms to gather community input and continuously improve communication efforts. | Equity-focused communication strategies ensure that all community members are informed, engaged, and respected, leading to more effective and inclusive health equity initiatives. | Grogan (2022), Calancie et al. (2022), Eslava-Schmalbach et al. (2019) |
